# Targeted xCT‐mediated Ferroptosis and Protumoral Polarization of Macrophages Is Effective against HCC and Enhances the Efficacy of the Anti‐PD‐1/L1 Response

**DOI:** 10.1002/advs.202203973

**Published:** 2022-11-28

**Authors:** Bufu Tang, Jinyu Zhu, Yajie Wang, Weiqian Chen, Shiji Fang, Weiyang Mao, Ziwei Xu, Yang Yang, Qiaoyou Weng, Zhongwei Zhao, Minjiang Chen, Jiansong Ji

**Affiliations:** ^1^ Key Laboratory of Imaging Diagnosis and Minimally Invasive Intervention Research Institute of Imaging Diagnosis and Minimally Invasive Intervention Research The Fifth Affiliated Hospital of Wenzhou Medical University Lishui Hospital of Zhejiang University Lishui 323000 China; ^2^ Clinical College of The Affiliated Central Hospital School of Medicine Lishui University Lishui 323000 China

**Keywords:** system x_c_^‐^, hepatocellular carcinoma (HCC), tumor‐associated macrophages (TAMs), GPX4, RRM2, anti‐tumor immunity

## Abstract

Tumor‐associated macrophages (TAMs) play an essential role in tumor progression, metastasis, and antitumor immunity. Ferroptosis has attracted extensive attention for its lethal effect on tumor cells, but the role of ferroptosis in TAMs and its impact on tumor progression have not been clearly defined. Using transgenic mouse models, this study determines that xCT‐specific knockout in macrophages is sufficient to limit tumorigenicity and metastasis in the mouse HCC models, achieved by reducing TAM recruitment and infiltration, inhibiting M2‐type polarization, and activating and enhancing ferroptosis activity within TAMs. The SOCS3‐STAT6‐PPAR‐*γ* signaling may be a crucial pathway in macrophage phenotypic shifting, and activation of intracellular ferroptosis is associated with GPX4/RRM2 signaling regulation. Furthermore, that xCT‐mediated macrophage ferroptosis significantly increases PD‐L1 expression in macrophages and improves the antitumor efficacy of anti‐PD‐L1 therapy is unveiled. The constructed Man@pSiNPs‐erastin specifically targets macrophage ferroptosis and protumoral polarization and combining this treatment with anti‐PD‐L1 exerts substantial antitumor efficacy. xCT expression in tumor tissues, especially in CD68+ macrophages, can serve as a reliable factor to predict the prognosis of HCC patients. These findings provide further insight into targeting ferroptosis activation in TAMs and regulating TAM infiltration and functional expression to achieve precise tumor prevention and improve therapeutic efficacy.

## Graphical abstract

xCT‐specific knockout in macrophages effectively limits tumorigenesis and metastasis in mice HCC models by reducing TAM recruitment and infiltration, inhibiting M2‐type polarization, and facilitating ferroptosis activity within TAMs, in which SOCS3‐STAT6‐PPAR‐*γ* signaling may be a crucial regulating pathway. xCT‐mediated macrophage ferroptosis significantly increases PD‐L1 expression in macrophages and combining Man@pSiNPs‐erastin targeted treatment with anti‐PD‐L1 exert substantial antitumor efficacy.

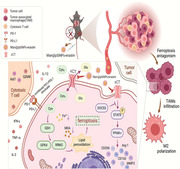

## 1 Introduction

As one of the leading threats to human health, liver cancer caused 905 677 new cases and 830 180 cancer‐related deaths worldwide in 2020, according to World Health Organization (WHO) statistics (https://gco.iarc.fr/today/fact‐sheets‐cancers).^[^ ^1^ ^]^ The overall 5‐year survival of liver cancer is ≈18%.^[^ ^2^ ^]^ Hepatocellular carcinoma (HCC), which represents ≈90% of primary liver cancers, is a highly heterogeneous disease^[^ ^3^ ^]^ whose carcinogenesis and progression closely correlate with complex and unique molecular pathogenesis, the tumor microenvironment (TME) and specific genetic factors.^[^ ^4^, ^5^ ^]^ In‐depth exploration and understanding of the molecular mechanisms and TME regulation in HCC are beneficial for improving our current understanding of the treatment of this disease and can provide patients with more effective individualized treatment options and improve disease outcomes.

The TME is considered to be the “fertile soil” in tumor initiation, development, and metastasis and plays an essential role in therapeutic responses and clinical outcomes.^[^ ^6^ ^]^ Tumor‐associated macrophages (TAMs), which are the most well‐defined tumor‐infiltrating immune cell type in the TME, play a prominent role in this process from early carcinogenesis to tumor progression.^[^ ^7^ ^]^ TAMs exhibit high plasticity in the TME, ranging from proinflammatory and antitumor (M1‐like) to anti‐inflammatory and protumoral (M2‐like) states. Substantial evidence has revealed that TAMs acquire a protumoral phenotype to support tumorigenesis, angiogenesis, and disease progression.^[^ ^8^ ^]^ Immunologically, the protumoral phenotype inhibits cytotoxic T lymphocyte (CTL) responses and regulates immunosuppression, which blocks effective antitumor immunity and immunotherapy, thereby leading to poor clinical prognosis.^[^ ^9^ ^]^ Notably, the expression of programmed cell death ligand 1 (PD‐L1) is a crucial mechanism in suppressing cytotoxic T lymphocyte (CTL) function to induce immune tolerance and facilitate tumor escape from the immune system.^[^ ^10^ ^]^ PD‐L1 can also be expressed by TAMs.^[^ ^11^ ^]^ To some extent, the presence of TAMs is an obstacle to targeted anticancer therapy and immunotherapy, but from another perspective, the targeted regulation of TAMs is also promising to achieve the goals of tumor prevention and enhance the efficacy of immunotherapy.

In recent years, increased evidence has pointed out the potential of triggering ferroptosis, a new programmed nonapoptotic mode of cell death characterized by the lethal accumulation of iron‐dependent lipid reactive oxygen species (ROS),^[^ ^12^ ^]^ in cancer development and suppression.^[^ ^13^, ^14^ ^]^ It is worth noting that there is an interaction between ferroptosis and the TME. A recent study revealed that oxidative stress induced the release of the KRAS^G12D^ protein from cancer cells that succumbed to autophagy‐dependent ferroptosis to the TME, which in turn drove the polarization of macrophages in the TME to the M2 phenotype to promote the growth of pancreatic cancer.^[^ ^15^ ^]^ In a previous study, we found that cancer cell ferroptosis influenced macrophage polarization in the TME.^[^ ^16^ ^]^ Nevertheless, the specific impact of macrophage‐mediated ferroptosis on the TME, especially on TAM infiltration and polarization, has not yet been fully clarified. Examining the regulatory effect of ferroptosis on TAMs may be a reliable entry point to provide new effective anticancer strategies for treating HCC.

xCT (encoded by SLC7A11) is the heavy chain subunit of the system x_c_^−^, which mediates the reverse transport of extracellular L‐cystine and intracellular L‐glutamic acid for intracellular glutathione biosynthesis and antioxidant defense to balance the enhanced oxidative stress in tumor cells and thereby meet the needs for rapid growth and development.^[^ ^17^ ^]^ xCT is essential for the survival, growth, and malignant progression of tumor cells and has been shown to be significantly upregulated in many human cancers.^[^ ^18^, ^19^ ^]^ What needs attention is that the promotion of SLC7A11 overexpression on tumor growth and progression are partly achieved by suppressing ferroptosis,^[^ ^19^, ^20^ ^]^ and some studies in recent years revealed that therapeutic strategies such as immunotherapy and radiotherapy induced cancer cell ferroptosis to enhance tumor suppression by modulating the expression of SLC7A11.^[^ ^21^, ^22^ ^]^ However, the role of ferroptosis in TME components, especially TAMs, which are key components of the TME, needs further examination and elucidation.

In addition to the known lethal effect caused by the activation of ferroptosis in tumor cells, in this study, we focused on examining whether activated ferroptosis in TAMs, a key component of the TME, is a crucial contributor that regulates the TME and enhances tumor suppression. By altering xCT expression, we modulated the activation of ferroptosis in TAMs and the infiltration and polarization of TAMs to examine their role in tumor progression and clarify the corresponding regulatory mechanism, aiming to provide new ideas and options for developing promising treatment strategies for HCC.

## 2 Results

### 2.1 TAMs Manifested xCT Upregulation, Ferroptosis Reduction, and M2‐Like Polarization

**Figure 1:**
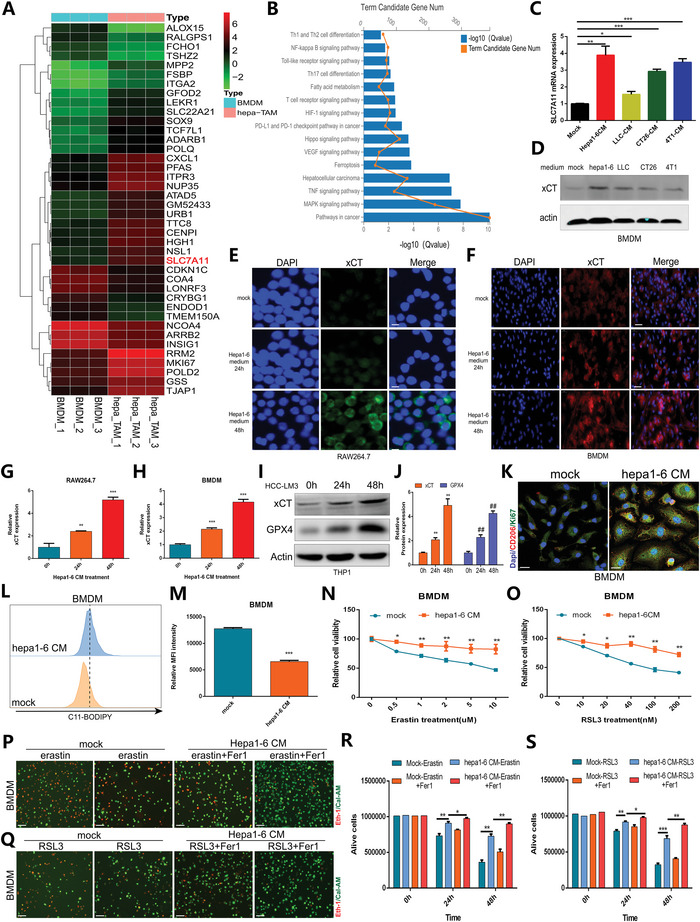
Characteristics of xCT expression, ferroptosis, and macrophage polarization in Hepa1‐6 CM‐cultured BMDMs. A) Heatmap showing the hierarchical clustering of DEGs between TAMs and BMDMs from WT mice (*n* = 3). B) KEGG functional analysis exhibiting the enriched signaling pathways associated with the DEGs. C) Expression characteristics of SLC7A11 in BMDMs cultured with CM from different tumor cells. D) Western blot showing the expression level of xCT in BMDMs cultured with CM from different tumor cells. E–H) IF results and quantification showing changes in xCT activity in RAW264.7 cells (E,G) and BMDMs (F,H) after being cultured with Hepa1‐6 CM. I,J) Western blot and quantification showing the expression of GPX4 in HCC‐LM3 CM‐cultured THP1 cells. K) Double IF staining showing the proportion of CD206+ Ki67+ macrophages in Hepa1‐6 CM‐cultured BMDMs. L,M) FCM with C11‐BODIPY (a marker of lipid peroxidation) probe and quantification showing decreased lipid peroxidation accumulation in Hepa1‐6 CM‐cultured BMDMs. N,O) The viability of BMDMs cultured with normal medium or Hepa1‐6 CM under erastin or RSL3 treatment is shown. P–S) Live/dead cell staining showing the cell viability of BMDMs cultured with normal medium or Hepa1‐6 CM under erastin or RSL3 treatment in a time course. All data in the figure are represented as the means ± SEM of three independent experiments. (C,G,H,J,Q,R) 1‐way ANOVA with Tukey's Multiple Comparison test. The result was normalized according to the result of Mock or 0 h. M–O) Student's *t*‐test. * *p* < 0.05, ** *p* < 0.01, *** *p* < 0.001.

To define the influence of xCT in TAMs on HCC progression, we first performed fluorescence‐activated cell sorting (FACS) to sort TAMs from Hepa1‐6 tumor‐bearing wild‐type (WT) mice models and performed RNA sequencing to explore the expression characteristics of differentially expressed genes (DEGs) between TAMs and bone marrow‐derived macrophages (BMDMs) from WT mice. We found that the expression of SLC7A11 in TAMs was significantly upregulated compared to that in BMDMs (**Figure** **1**A). Subsequent Kyoto Encyclopedia of Genes and Genomes (KEGG) analysis revealed that TAM DEGs may function in ferroptosis (Figure 1B). Then, we cultured BMDMs with conditional medium (CM) from different tumor cells, and the quantitative reverse transcription‐polymerase chain reaction (qRT‐PCR) and western blot (WB) results revealed significant upregulation of xCT, especially in Hepa1‐6 CM‐cultured BMDMs (Figure 1C,D). We further performed an IF assay and confirmed that xCT expression was increased in both RAW264.7 cells and BMDMs with incubation time in the presence of Hepa1‐6 CM (Figure 1E–H). To evaluate ferroptosis activity in macrophages, we detected the expression of the ferroptosis suppressor gene GXP4 and found that its expression in human THP1 macrophages increased with incubation time in the presence of HCC‐LM3 CM (Figure 1I,J). We also examined the phenotypic shift in macrophages and confirmed that proliferating BMDMs were mainly polarized to the M2‐like phenotype after being cultured in Hepa1‐6 CM (Figure 1K). In addition, the decrease in lipid peroxidation accumulation in Hepa1‐6 CM‐cultured BMDMs shown by flow cytometry (FCM) was consistent with the changes in xCT expression (Figure 1L,M). Then, we treated BMDMs that were cultured with normal medium or Hepa1‐6 CM using the ferroptosis inducer erastin or RSL3, and the results showed that Hepa1‐6 CM‐cultured BMDMs exhibited stronger viability (Figure 1N,O), and the cellular lethal effect induced by erastin or RSL3 was effectively rescued under ferroptosis inhibitor Fer‐1 treatment (Figure 1P–S). The results suggested that Hepa1‐6 CM‐cultured BMDMs had reduced ferroptosis activation, thereby suppressing the cellular lethal effect induced by ferroptosis. Based on these findings, we hypothesized that there may be a correlation between the increased expression of xCT and M2‐like macrophage polarization, as well as a reduction in ferroptosis in TAMs.

### 2.2 xCT‐Modulate HCC Tumorigenesis Correlates with Macrophage Infiltration

**Figure 2:**
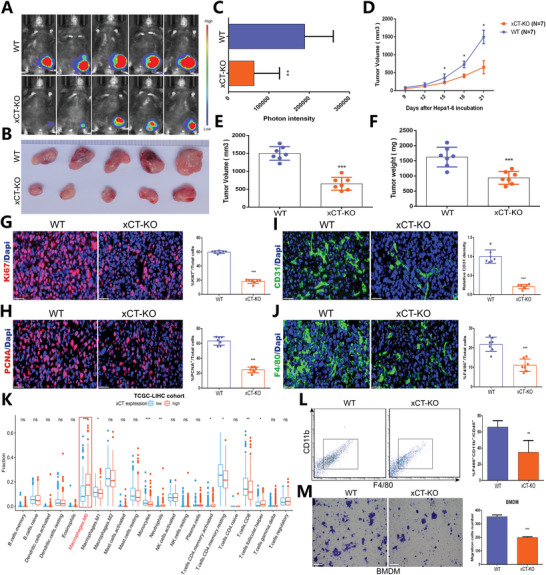
The inhibitory effect of xCT KO on HCC tumorigenesis is associated with macrophage infiltration. A) In vivo bioluminescence imaging detecting the development of subcutaneous Hepa1‐6 tumors in WT mice and xCT‐KO mice (*n* = 5/group). B) Difference in the growth of Hepa1‐6 tumors in WT mice and xCT‐KO mice (*n* = 5). C) Photon intensity of Hepa1‐6 tumors in WT mice and xCT‐KO mice (*n* = 5). D–F) Differences in tumor volume (D,E) and tumor weight (F) in WT mice and xCT‐KO mice. G–J) IF results showing the expression of Ki67 (G), PCNA (H), CD31 (I) and F4/80 (J) in Hepa1‐6 tumor tissues from WT mice and xCT‐KO mice. The result of (I) was normalized according to the result of xCT^f/f^. K) Correlation of immune cell infiltration in the TME and xCT expression from TCGA‐LIHC cohort. L) FCM analysis of TAM infiltration in Hepa1‐6 tumors in WT mice and xCT‐KO mice (*n* = 3). M) Transwell assay showing the migration capability of BMDMs derived from WT mice and xCT‐KO mice (*n* = 3). All data in the figure are represented as the means ± SEM. Differences between the groups were evaluated using Student's *t*‐test. ns, no significance, * *p* < 0.05, ** *p* < 0.01, *** *p* < 0.001, **** *p* < 0.0001.

As a major component of the TME, TAMs strongly contribute to tumor development and progression,^[^ ^32^ ^]^ and we found that xCT expression levels in TAMs were significantly elevated, we speculated that xCT may be involved in the recruitment and infiltration of TAMs and thereby affect the progression of HCC. To investigate this hypothesis, we first constructed xenograft models by subcutaneously injecting Hepa1‐6 cells into WT mice and xCT‐KO mice. Genotyping analysis and xCT knockout confirmation to identify the mice of different genotypes is exhibited in Figure S1, Supporting Information. As shown, tumors were significantly smaller in xCT‐KO mice than in WT mice (**Figure** **2**A–C). In addition, xCT‐KO mice exhibited significantly attenuated tumor growth and reduced tumor weights compared to WT mice (Figure 2D–F). The IF results revealed that the expression levels of proliferation markers, including Ki67 and PCNA, in WT tumors was significantly higher than that in xCT‐KO tumors (Figure 2G,H). CD31 was used as a blood vessel marker, and the blood vessel area was significantly reduced in xCT‐KO tumors compared with WT tumors (Figure 2I). These findings implied that xCT KO hampered the growth and development of HCC.

Subsequently, we examined the effect of xCT KO on macrophage infiltration. We first assessed the effect of xCT on monocyte phenotypes and it was determined that xCT expression did not cause the alteration in terms of the frequency of Ly6C^high^, Ly6C^mid^, and Ly6C^low^ monocytes (Figure S2, Supporting Information). Then we used the murine macrophage marker F4/80 and found that the inhibitory effects of xCT KO on tumor development were accompanied by a massive decrease in the proportion of infiltrating macrophages (Figure 2J). By analyzing the link between xCT and immune cells in the TME from TCGA‐LIHC cohort (Figure 2K) and ICGC database (Figure S3, Supporting Information), we also found that macrophage infiltration was closely related to xCT expression, and subsequent FCM further revealed that the number of F4/80+ CD11b+ macrophages was substantially reduced in xCT‐KO tumors compared to WT tumors (Figure 2L). In addition, transwell assays confirmed that xCT KO attenuated the migration of BMDMs (Figure 2M). These results indicated that the suppressive effect of xCT KO on tumor growth may be mediated partly through reducing the infiltration of TAMs and inhibiting their protumorigenic effects.

### 2.3 Macrophage‐Derived xCT Facilitates Carcinogenesis and Spontaneous Lung Metastasis of HCC

**Figure 3:**
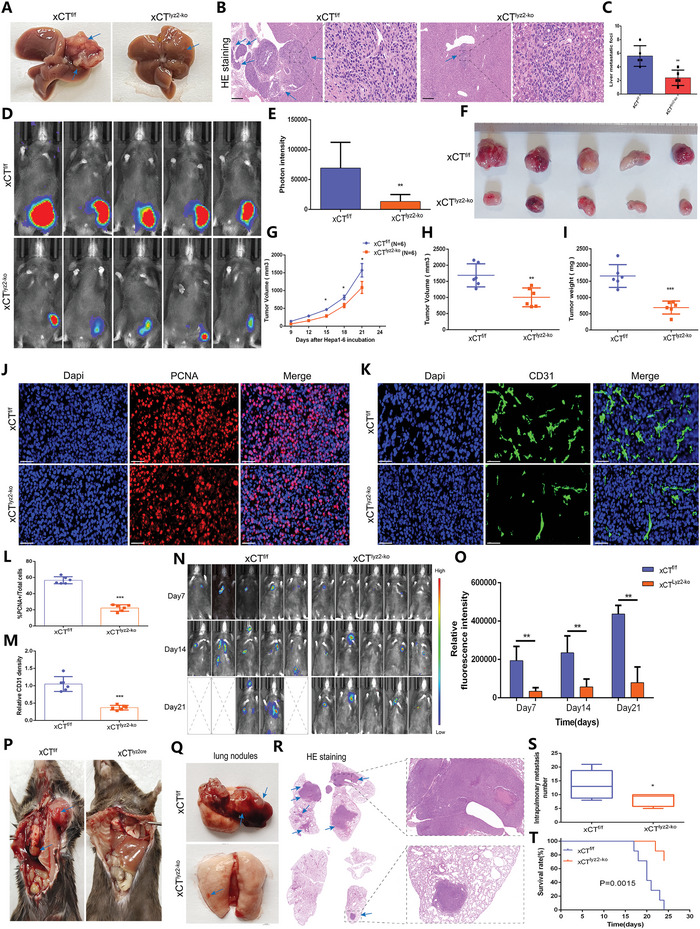
Macrophage‐derived xCT contributes to the carcinogenesis and metastasis of HCC. A) Macroscopic changes in liver carcinogenesis in hydrodynamic tail vein injection models. B) HE staining showing pathological changes in the liver tissues in hydrodynamic tail vein injection models. C) Liver oncogenic foci in the hydrodynamic tail vein injection models (*n* = 5). D) In vivo bioluminescence imaging showing of subcutaneous Hepa1‐6 tumors in xCT^f/f^ mice and xCT^lyz2cre^ mice (*n* = 5/group). E) Photon intensity of tumors in xCT^f/f^ mice and xCT^lyz2cre^ mice (*n* = 5). F) Differences in tumor growth in xCT^f/f^ mice and xCT^lyz2cre^ mice (*n* = 5). G–I) Differences in tumor volume (G,H) and tumor weight (I) between xCT^f/f^ mice and xCT^lyz2cre^ mice (*n* = 5). J–M) IF staining and quantification showing the expression of PCNA (J,L) and CD31 (K,M) in Hepa1‐6 tumors from xCT^f/f^ mice and xCT^lyz2cre^ mice. The result of (L) was normalized according to the result of xCT^f/f^. N) In vivo bioluminescence imaging showing lung metastasis in xCT^f/f^ mice and xCT^lyz2cre^ mice after caudal vein injection of Hepa1‐6 cells (*n* = 5). O) Relative fluorescence intensity of lung metastasis in xCT^f/f^ mice and xCT^lyz2cre^ mice (*n* = 5). P,Q) Macroscopic changes in lung metastasis in xCT^f/f^ mice and xCT^lyz2cre^ mice. R) HE staining showing pathological changes in the lung tissues in xCT^f/f^ mice and xCT^lyz2cre^ mice. S) Intrapulmonary metastasis numbers in xCT^f/f^ mice and xCT^lyz2cre^ mice (*n* = 6). T) K–M curves showing the survival rate of xCT^f/f^ mice and xCT^lyz2cre^ mice (*n* = 6). All data in the figure are represented as the means ± SEM. Differences between the groups were evaluated using Student's *t*‐test. * *p* < 0.05, ** *p* < 0.01, *** *p* < 0.001.

To further characterize the functional link between xCT and TAMs, we evaluated the effect of macrophage‐specific xCT KO on tumor growth and metastasis. We constructed hydrodynamic tail vein injection models of HCC by rapidly injecting Hepa1‐6 cells into the tail vein of xCT^f/f^ mice and xCT^lyz2cre^ mice within 5–7 s. As shown in **Figure** **3**A–C and Figure S4A, Supporting Information, comparing to xCT^f/f^ mice, the tumor carcinogenic capability was significantly attenuated in xCT^lyz2cre^ mice. There was no tumor growth in other organs including heart, lung, kidney, and spleen (Figure S4B, Supporting Information). We then also constructed other transplantable HCC models by subcutaneously injecting Hepa1‐6 cells into xCT^f/f^ mice and xCT^lyz2cre^ mice. Tumor growth was also significantly reduced in xCT^lyz2cre^ mice compared with xCT^f/f^ mice (Figure 3D–F). Furthermore, decreased tumor volume and tumor weight were also observed in xCT^lyz2cre^ mice (Figure 3G–I). IF staining revealed significant reductions in PCNA and CD31 levels in xCT^lyz2cre^ tumors compared to xCT^f/f^ tumors (Figure 3J–M). These results indicated that specific ablation of xCT in macrophages was sufficient to suppress tumor development.

Then, we further established HCC lung metastasis mouse models through caudal vein injection of Hepa1‐6 cells. As shown by in vivo bioluminescence imaging, tumor lung metastasis was more aggressive in xCT^f/f^ mice than in xCT^lyz2cre^ mice (Figure 3N,O). Macroscopic changes and HE staining further confirmed the attenuated metastasis capability of tumors in xCT^lyz2cre^ mice compared to xCT^f/f^ mice (Figure 3P–R). Figure 3S reveals a significant reduction in intrapulmonary metastasis numbers in xCT^lyz2cre^ mice compared to xCT^f/f^ mice. In addition, survival analysis indicated that xCT^lyz2cre^ mice exhibited a more positive survival advantage than xCT^f/f^ mice (Figure 3T). In summary, these findings highlight the crucial role of specific ablation of xCT in macrophages in suppressing tumor growth and metastasis.

### 2.4 xCT Was Involved in the Activation of SOCS3‐STAT6‐PPAR‐*γ* Signaling Pathway and Regulated the M2‐Like Polarization of Macrophages

**Figure 4:**
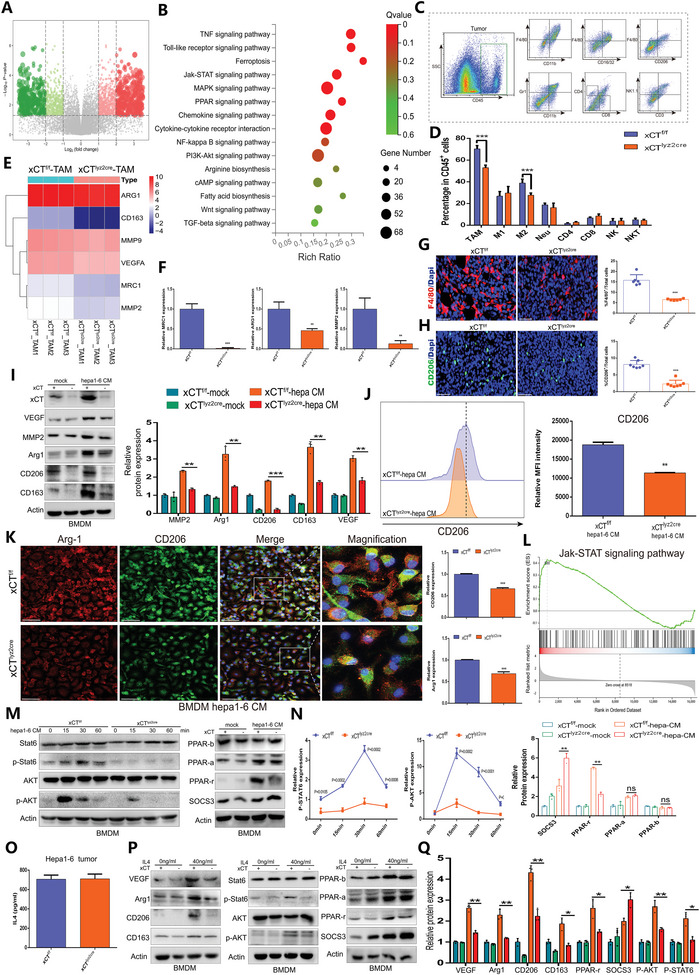
The SOCS3‐STAT6‐PPAR‐*γ* signaling pathway is involved in the M2‐like polarization of macrophages via xCT. A) Volcano plot depicting the expression characteristics of DEGs in TAMs from xCT^f/f^ mice and xCT^lyz2cre^ mice. B) KEGG functional analysis showing the enriched signaling pathways associated with the DEGs. C,D) FACS analysis and quantification showing the percentage of immune cells in single‐cell suspensions of Hepa1‐6 tumors from xCT^f/f^ mice and xCT^lyz2cre^ mice (*n* = 5). E) Heatmap exhibiting the expression profiles of M2‐like markers in TAMs from xCT^f/f^ mice and xCT^lyz2cre^ mice (*n* = 3). F) qRT‐PCR analysis revealing the differences in the expression of MRC1, ARG1, and MMP2 in TAMs from xCT^f/f^ mice and xCT^lyz2cre^ mice (*n* = 3). G,H) IF staining showing the expression of F4/80 (G) and CD206 (H) in Hepa1‐6 tumor tissues from xCT^f/f^ mice and xCT^lyz2cre^ mice (*n* = 7). I) Western blot evaluating the expression of M2‐like macrophage‐related factors in BMDMs from xCT^f/f^ mice and xCT^lyz2cre^ mice cultured with or without Hepa1‐6 CM (*n* = 3). J) FCM histogram profiles depicting the fluorescence intensity of CD206 in Hepa1‐6 CM‐cultured BMDMs from xCT^f/f^ mice and xCT^lyz2cre^ mice (*n* = 3). K) IF staining showing the proportion of infiltrating Arg‐1+ CD206+ macrophages in Hepa1‐6 CM‐cultured BMDMs from xCT^f/f^ mice and xCT^lyz2cre^ mice (*n* = 3). L) GSEA indicating a significant change in the Jak‐STAT signaling pathway in BMDMs from xCT^lyz2cre^ mice. M,N) Western blot showing the impact of xCT expression on the activity of the SOCS3‐STAT6‐PPAR‐*γ* axis in Hepa1‐6 CM‐cultured BMDMs (*n* = 3). O) ELISA analysis showing the IL‐4 concentration in co‐culture systems of Hepa 1–6 cells and BMDMs from xCT^f/f^ or xCT^Lyz2Cre^ mice (*n* = 3). P,Q) Western blot showing the effect of xCT expression on the activity of the IL‐4 mediated SOCS3‐STAT6‐PPAR‐*γ* signaling. The result was normalized according to the result of xCT^f/f^. All data in the figure are represented as the means ± SEM. Differences between the groups were evaluated using Student's *t*‐test. ns, no significance, ** *p* < 0.01, *** *p* < 0.001.

As described above, specific ablation of xCT in macrophages significantly attenuated the progression and metastasis of tumors, implying that xCT KO in macrophages may result in functional alterations in macrophages and/or impair their crosstalk with tumor cells. To validate these hypotheses, we performed RNA sequencing of TAMs sorted from xCT^f/f^ tumors and xCT^lyz2cre^ tumors and identified the DEGs (**Figure** **4**A). KEGG analysis of the DEGs revealed that xCT^lyz2cre^ TAMs mainly involved functions and pathways, including ferroptosis, the Jak‐STAT signaling pathway and the PPAR signaling pathway (Figure 4B). Then, we performed FCM to examine the effect of xCT expression on the tumor immune subpopulation, and the results showed significantly decreased infiltration of TAMs and M2 macrophages in xCT^lyz2cre^ tumors compared to xCT^f/f^ tumors (Figure 4C,D).

To better corroborate the phenotypic shift in xCT^lyz2cre^ TAMs, we measured the expression of M2‐like markers, and the results revealed a substantial reduction in MRC1, ARG1, MMP9 and MMP2 expression in xCT^lyz2cre^ TAMs compared with xCT^f/f^ TAMs (Figure 4E,F). These secreted factors are also known to mediate the crosstalk between tumor cells and macrophages.^[^ ^33^ ^]^ The IF results showed that the proportion of infiltrating F4/80+ macrophages and CD206+ macrophages was significantly reduced in xCT^lyz2cre^ tumors compared to xCT^f/f^ tumors (Figure 4G,H). The WB results further confirmed that the enhanced M2‐like phenotype polarization of BMDMs cultured with Hepa1‐6 CM was significantly suppressed by specific xCT KO in macrophages (Figure 4I). Consistently, FCM revealed a significant reduction in CD206+ macrophages in Hepa1‐6 CM‐cultured BMDMs from xCT^f/f^ mice compared to Hepa1‐6 CM‐cultured BMDMs from xCT^lyz2cre^ mice (Figure 4J). Double IF staining for ARG1 and CD206 further confirmed that the proportion of the M2‐like phenotype was significantly reduced in xCT^lyz2cre^ TAMs compared to xCT^f/f^ TAMs (Figure 4K).

GSEA highlighted changes in the Jak‐STAT signaling pathway in xCT^lyz2cre^ TAMs, which may influence the polarization of macrophages (Figure 4L). To validate the impact of xCT on this pathway, we assessed the expression of effectors in the Jak‐STAT signaling pathway and confirmed that xCT KO in macrophages significantly suppressed the activity of phospho‐STAT6, phospho‐AKT and PPAR‐*γ* while significantly increasing the expression of suppressor of cytokine signaling 3 (SOCS3) (Figure 4M,N). Considering that IL‐4 is a well‐known pro‐M2 polarizing factor, we further detected the expression of IL‐4 in co‐culture systems of Hepa 1–6 cells and BMDMs from xCTf/f or xCTLyz2Cre mice and found there were no significant difference on IL‐4 concentration between the two co‐culture systems (Figure 4O). Then we treated BMDMs with IL‐4 and found xCT KO in macrophages markedly suppressed IL‐4‐mediated SOCS3‐STAT6‐PPAR‐*γ* pathway, as well as the expression of factors including Arg1, CD206 and CD163(Figure 4P,Q), suggesting that xCT KO disrupted macrophage responsiveness to IL4 in tumor microenvironment, but not the production of IL4 cytokine. These results indicated that xCT served as a positive regulator of IL‐4 mediated M2 polarization of macrophages and affected the activity of SOCS3‐STAT6‐PPAR‐*γ* signaling axis, which may be a crucial pathway involved in a protumorigenic M2‐like phenotype shift in the TME.

### 2.5 Ferroptosis Induction in the Context of Macrophage xCT Knockout Hampers the Progression of HCC

**Figure 5:**
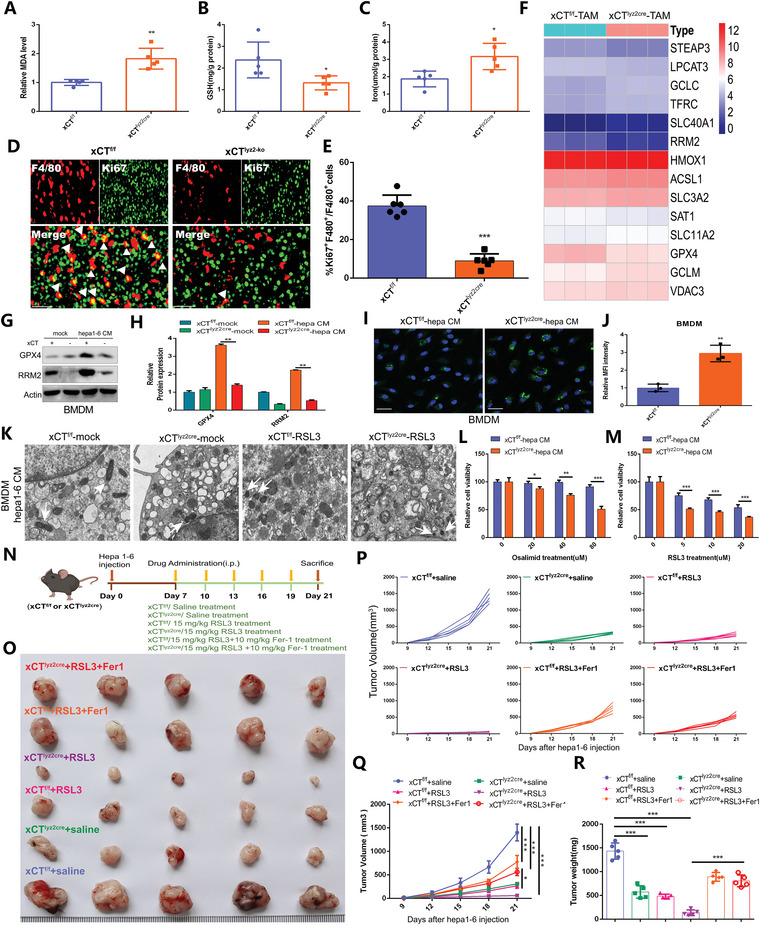
Macrophage‐specific xCT knockout triggers ferroptosis to suppress the progression of HCC. A–C) Expression of MDA (A), GSH (B), and iron (C) in xCT^f/f^ TAMs and xCT^lyz2cre^ TAMs (*n* = 5). The result of (A) was normalized according to the result of xCT^f/f^. D,E) IF staining (D) and quantification (E) showing the infiltration of F4/80+ Ki67+ macrophages in Hepa1‐6 tumors from xCT^f/f^ mice and xCT^lyz2cre^ mice (*n* = 6). F) Heatmap revealing the expression characteristic of ferroptosis‐related genes in xCT^f/f^ TAMs and xCT^lyz2cre^ TAMs (*n* = 3). G,H) Western blot (G) and quantification (H) showing the expression of GPX4 and RRM2 in BMDMs from xCT^f/f^ mice and xCT^lyz2cre^ mice cultured with or without Hepa1‐6 CM (*n* = 3). The result was normalized according to the result of xCT^f/f^‐mock. I,J) IF staining with C11‐BODIPY probe (I) and quantification (J) exhibiting the difference of lipid peroxidation accumulation in Hepa1‐6 CM‐cultured BMDMs from xCT^f/f^ mice and xCT^lyz2cre^ mice (*n* = 3). The result was normalized according to the result of xCT^f/f^. K) Representative TEM images of mitochondria in Hepa1‐6 CM‐cultured BMDMs from xCT^f/f^ mice and xCT^lyz2cre^ mice with or without RSL3 treatment. L,M) Effect of Osalimid (L) and RSL3 (M) treatment on the viability of Hepa1‐6 CM‐cultured BMDMs from xCT^f/f^ mice and xCT^lyz2cre^ mice (*n* = 3). N) Treatment scheme for Hepa1‐6 tumor‐bearing mouse models. O) The growth of Hepa1‐6 tumors in response to different treatments (*n* = 5/group). P,Q) Differences in tumor volume in response to different treatments (*n* = 5). R) Differences in tumor weight in response to different treatments (*n* = 5). All data in the figure are represented as the means ± SEM. A–C,E,J,L,M) Student's *t*‐test. H,Q,R) 1‐way ANOVA with Tukey's Multiple Comparison test. * *p* < 0.05, ** *p* < 0.01, *** *p* < 0.001.

The correlation of xCT KO in TAMs with ferroptosis as mentioned above (Figures 1 and 4B) and the known lethal effects of ferroptosis triggering on cells prompted us to further investigate the effect of xCT expression on ferroptosis in TAMs. Compared with that in the xCT^f/f^ TAMs, substantially increased levels of ferroptosis‐related indicators, including MDA and iron, and significantly reduced levels of GSH were detected in xCT^lyz2cre^ TAMs (**Figure** **5**A–C). Double IF staining for F4/80 and Ki67 indicated that the infiltration of proliferative TAMs in xCT^lyz2cre^ tumors was much lower than that in xCT^f/f^ tumors, implying that xCT KO in TAMs promoted intracellular ferroptosis and thereby suppressed TAM infiltration (Figure 5D,E).

To gain insight into the underlying mechanism of the enhanced ferroptosis activity in xCT^lyz2cre^ TAMs, we evaluated the expression of ferroptosis‐regulated factors in xCT^lyz2cre^ TAMs and xCT^f/f^ TAMs (Figure 5F). WB analysis showed that the upregulation of glutathione peroxidase 4 (GPX4) and ribonucleotide reductase regulatory subunit M2 (RRM2) in BMDMs cultured with Hepa1‐6 CM was blocked by xCT KO (Figure 5G,H). In addition, xCT KO significantly increased the accumulation of lipid peroxidation in BMDMs cultured with Hepa1‐6 CM (Figure 5I,J). Transmission electron microscopy (TEM) images of Hepa1‐6 CM‐cultured BMDMs further determined that intracellular ferroptosis was triggered by xCT KO and exacerbated by RSL3 treatment (Figure 5K). As shown, in response to the same dose of Osalimid (an inhibitor of RRM2) and RSL3, the viability of Hepa1‐6 CM‐cultured BMDMs from WT mice was stronger than that of cells from xCT‐KO mice, indicating that xCT KO effectively enhanced intracellular ferroptosis activity. These results indicated that ferroptosis activation was related to GPX4/RRM2 signaling. RRM2 may act as a potential ferroptosis suppressor, and its inhibitor Osalimid may act as a targeted ferroptosis inducer (Figure 5L,M). Subsequently, we used xCT^lyz2cre^ mice and xCT^f/f^ mice to construct Hepa1‐6 tumor‐bearing models and divided the mice into 6 groups, which were treated with saline, RSL3 or RSL3 plus the Fer‐1 (5 mice/group) (Figure 5N). It was further revealed that xCT^lyz2cre^ mice had attenuated tumor growth and that the inhibitory effect was enhanced by RSL3 treatment, but notably, the inhibitory effect of macrophage‐specific xCT KO and RSL3 treatment on tumor growth was partly rescued by Fer‐1 (Figure 5O). Figure 5P–R and Figure S5A, Supporting Information, show the changes in tumor volume and tumor weight in response to different treatments. The changes in body weight in response to different treatments showed no significant differences (Figure S5B, Supporting Information) and HE staining indicated these treatments barely lead to organs toxicity in vivo (Figure S5C, Supporting Information). These results implied that xCT‐mediated macrophage ferroptosis trigger and activity enhancement within TAMs leads to tumor growth blockade, which is associated with GPX4/RRM2 signaling regulation.

### 2.6 xCT‐mediated Macrophage Ferroptosis and Polarization Enhance the Efficacy of Anti‐PD‐L1 Therapy Against HCC

**Figure 6:**
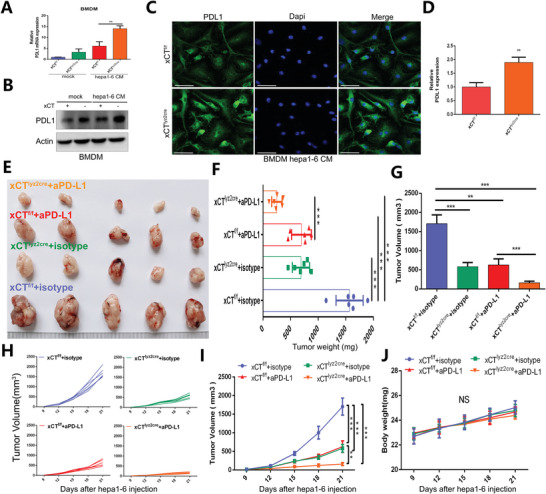
xCT‐mediated TAM ferroptosis and polarization promote the efficacy of PD‐1/PD‐L1 therapy against HCC. A,B) qRT‐PCR and Western blot showing the expression of PD‐L1 in xCT^f/f^ BMDMs and xCT^lyz2cre^ BMDMs cultured with normal medium or Hepa1‐6 CM (*n* = 3). The result of (A) was normalized according to the result of xCT^f/f^‐mock. C,D) IF staining and quantification exhibiting the expression characteristics of PD‐L1 in xCT^f/f^ tumor tissues and xCT^lyz2cre^ tumor tissues (*n* = 3). The result of was normalized according to the result of xCT^f/f^. E) The growth of Hepa1‐6 tumors in response to different treatments (*n* = 5). F–I) Differences in tumor weight (F), tumor load (G), and tumor volume (H,I) in response to different treatments (*n* = 6). J) Changes in mouse body weight in response to different treatments (*n* = 6). All data in the figure are represented as the means ± SEM. A,F,G,I,J) 1‐way ANOVA with Tukey's Multiple Comparison test. D) Student's *t*‐test. ns, no significance, ** *p* < 0.01, *** *p* < 0.001.

Since TAM infiltration and the protumoral phenotype are known to carry out a crucial impact on inducing immune tolerance and interfering with effective immunotherapy,^[^ ^34^ ^]^ and our previous finding revealed the close correlation between tumor cell ferroptosis with immune infiltration and to indicate the responsiveness of immunotherapy,^[^ ^35^ ^]^ we further focused on whether xCT‐mediated macrophage ferroptosis and polarization regulated the efficacy of immunotherapy. We examined PD‐L1 expression in BMDMs from xCT^f/f^ mice and xCT^lyz2cre^ mice that were cultured with normal medium or Hepa1‐6 CM, and the highest expression was found in xCT^lyz2cre^ BMDMs cultured with Hepa1‐6 CM (**Figure** **6**A). A consistent result was also confirmed by WB (Figure 6B), indicating that BMDMs increased PD‐L1 expression after culturing in Hepa1‐6 CM and that the upregulated expression of PD‐L1 was further promoted by xCT KO in macrophages. IF staining confirmed that PD‐L1 expression in xCT^lyz2cre^ tumor tissues was much higher than that in xCT^f/f^ tumor tissues (Figure 6C,D).

Then, we further explored antitumor efficacy in vivo with macrophage xCT KO and anti‐PD‐L1 treatment. xCT KO in macrophages or anti‐PD‐L1 treatment effectively attenuated tumor growth, and the combination of xCT KO in macrophages and anti‐PD‐L1 achieved the most robust tumor suppression (Figure 6E). The changes in tumor weight and tumor volume in response to different treatments was also consistent with the above findings (Figure 6F–I), while the changes in body weight in response to different treatments showed no significant differences (Figure 6J). These results suggested that xCT‐mediated macrophage ferroptosis and polarization effectively increased the expression of PD‐L1 in TAMs and strengthened the efficacy of anti‐PD‐L1 therapy against HCC.

### 2.7 The Constructed Nanoparticles Explicitly Target TAM Ferroptosis and Validate the Role of Ferroptosis in the TAM Phenotype

**Figure 7:**
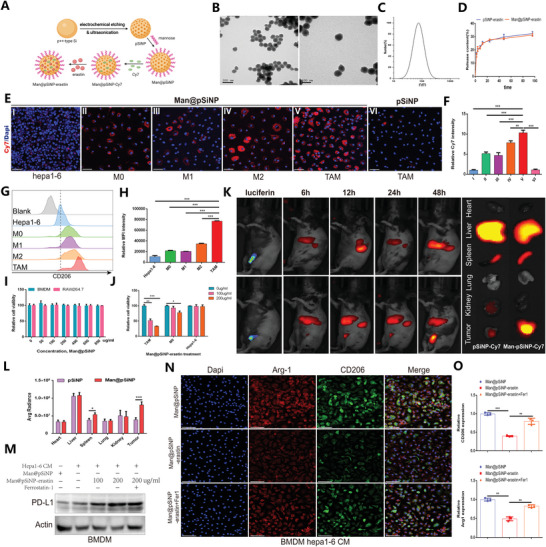
Man@pSiNP‐erastin specifically targets TAM ferroptosis and protumoral polarization in HCC. A) Schematic illustration of the preparation of pSiNPs. Created with Biorender.com. B) TEM images of pSiNPs. C) Particle size distribution of PSiNPs by DLS analysis. D) In vitro erastin release profiles of pSiNPs‐erastin and Man@pSiNPs‐erastin. E,F) IF staining revealing the specific targeting of Man@pSiNPs compared with pSiNPs (*n* = 3). The result of was normalized according to the result of Hepa 1–6. G) FCM histogram profiles of fluorescence intensity. H) Quantification of the mean fluorescence intensity by FCM analysis (*n* = 3). I) Cell viability results showing the potential toxicity of Man@pSiNPs on BMDMs and RAW264.7 cells (*n* = 3). J) The impact of Man@pSiNPs‐erastin treatment on the viability of TAMs, M0 cells, and Hepa1‐6 cells (*n* = 3). K,L) In vivo bioluminescence imaging and quantification showing the biodistribution of Man@pSiNPs‐Cy7 in mice (*n* = 5). M) Expression profiles of PD‐L1 in TAMs in response to different treatments. N,O) IF staining and quantification depicting the infiltration levels of Arg‐1+ macrophages and CD206+ macrophages in response to different treatments (*n* = 3). The result of was normalized according to the result of Man@pSINP. All data in the figure are represented as the means ± SEM. F,H,J,O,P) 1‐way ANOVA with Tukey's Multiple Comparison test. D,I,L) Student's *t*‐test. * *p* < 0.05, ** *p* < 0.01, *** *p* < 0.001.

Nanomaterials offer the potential for precision‐targeted molecular therapies in the clinic. Given that the phenotype of macrophages is often characterized by differences in the expression of various surface receptors, and CD206 macrophage mannose receptor is usually overexpressed on M2 polarized macrophages and TAMs, it is allowed to construct mannose modified nanocarrier to target M2 and TAMs.^[^ ^36^, ^37^ ^]^ In this study, we further explored and prepared mannose‐functionalized porous silicon nanoparticles (Man@pSiNPs), a functional nanocarrier that can specifically target TAMs. **Figure** **7**A depicts the schematic illustration of the Man@pSiNP preparation. TEM images showed that the particle size was homogenous (Figure 7B). DLS analysis showed that Man@pSiNPs were ≈100 nm in size (Figure 7C). Then, erastin was encapsulated by Man@pSiNPs by mixing erastin with the Man@pSiNPs in a PBS solution with stirring for 24 h at room temperature. Figure 7D indicates that pSiNPs‐erastin and Man@pSiNPs‐erastin exhibited a sustained drug release profile.

We first evaluated the specific targeting ability of Man@pSiNPs. The near infrared dye cyanine 7 (Cy7) was used to label Man@pSiNPs and porous silicon nanoparticles (pSiNPs) before administration. As shown, Man@pSiNPs‐Cy7 was specifically taken up by TAMs, especially M2‐like phenotype, while the accumulation of pSiNPs‐Cy7 in TAMs was very low (Figure 7E–H). Cell viability analysis suggested that Man@pSiNPs had no significant toxicity at the given concentrations on either BMDMs or RAW264.7 cells, revealing the excellent biocompatibility of Man@pSiNPs (Figure 7I). Then, we observed that Man@pSiNPs‐erastin showed dose‐dependent cytotoxicity, with a higher erastin concentration that resulted in more specific killing of TAMs and M0 cells but generated no negative effects on Hepa1‐6 cells, indicating that Man@pSiNPs‐erastin specifically targeted TAMs and inhibited cell viability by inducing the intracellular ferroptosis (Figure 7J). Furthermore, in vivo biodistribution experiments confirmed that at 48 h postinjection, Man@pSiNPs‐Cy7 were highly accumulated in tumors where TAMs were enriched, while the accumulation level of pSiNPs‐Cy7 in tumors was low (Figure 7K,L). In addition, both pSiNPs‐Cy7 and Man@pSiNPs‐Cy7 also accumulated highly in the liver and spleen, which is likely due to pSiNPs being trapped by Kupffer cells and splenic macrophages,^[^ ^38^ ^]^ and mannose functionalization enhanced the capture of pSiNPs by splenic macrophages. The expression of PD‐L1 was significantly dose‐dependent increased in TAMs treated with Man@pSiNPs‐erastin, while Fer‐1 partially reversed the upregulation effect of erastin on PD‐L1 expression (Figure 7M). The IF staining results suggested that Man@pSiNPs‐erastin significantly suppressed M2‐like polarization, but the inhibitory effect of erastin was partially rescued by Fer‐1 (Figure 7N,O). Overall, the prepared Man@pSiNPs acquired the ability of specifically targeting TAMs and achieved the intracellular ferroptosis activation and regulated TAM phenotype through the encapsulation of erastin.

### 2.8 Man@pSiNP Targeted Delivery of Ferroptosis Inducers Synergizes with Anti‐PD‐L1 Therapy in HCC

**Figure 8:**
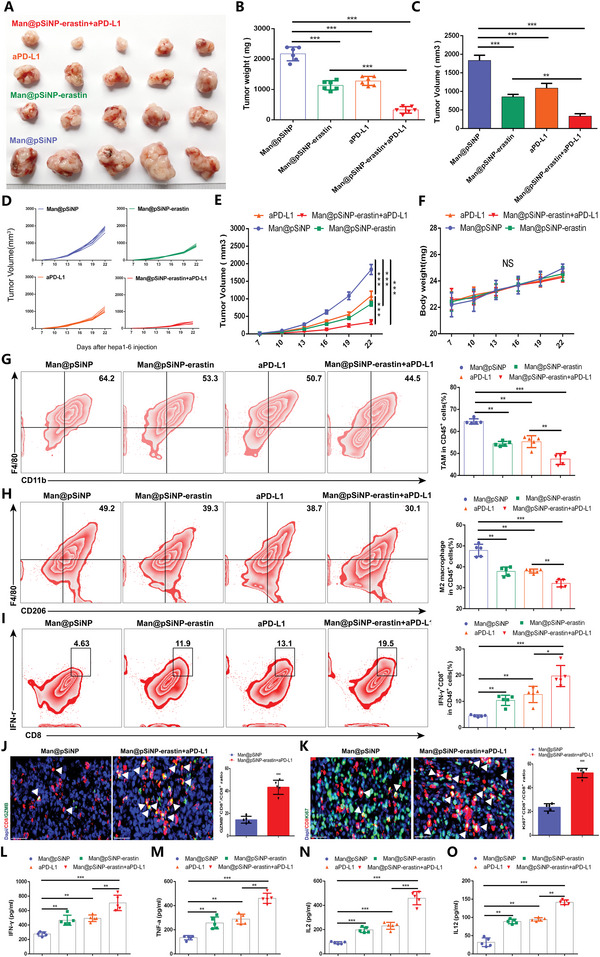
Man@pSiNP‐erastin synergizes with anti‐PD‐L1 therapy to suppress HCC. A) The growth of Hepa1‐6 tumors in response to Man@pSiNP‐erastin and/or anti‐PD‐L1 treatment (*n* = 6/group). B–E) Differences in tumor weight (B) and tumor volume (C–E) in response to different treatments (*n* = 6). F) Changes in mouse body weight in response to different treatments (*n* = 6). G–I) FCM analysis exhibiting the proportions of infiltrating TAMs, M2‐like cells, and IFN‐*γ* CD8^+^ cells in tumor tissues in response to different treatments (*n* = 5). J,K) IF staining depicting the infiltration of GZMB+ CD8^+^ cells and Ki67+ CD8^+^ cells in tumor tissues in response to combined treatment with Man@pSiNPs‐erastin and anti‐PD‐L1 (*n* = 5). L–O) The expression of immune‐related cytokines, including IFN‐*γ*, TNF‐*α*, IL‐2 and IL‐12, in response to different treatments (*n* = 5). All data in the figure are represented as the means ± SEM. B,C,E–I,L–O) 1‐way ANOVA with Tukey's Multiple Comparison test. J,K) Student's *t*‐test. ns, no significance, * *p* < 0.05, ** *p* < 0.01, *** *p* < 0.001.

To analyze the anticancer effect of Man@pSiNP‐erastin treatment combined with anti‐PD‐L1 therapy in vivo, Hepa1‐6 tumor‐bearing C57BL/6 male mice were randomly divided into four groups (6 mice/group) and treated with Man@pSiNP, Man@pSiNP‐erastin, anti‐PD‐L1, or a combination of Man@pSiNPs‐erastin and anti‐PD‐L1, separately. Man@pSiNP‐erastin or anti‐PD‐L1 treatment alone effectively attenuated tumor growth and development, and importantly, the combination of Man@pSiNPs‐erastin and anti‐PD‐L1 treatment achieved optimal anticancer efficacy (**Figure** **8**A–E). There was no significant difference among the body weights of mice after the different treatments, indicating that Man@pSiNPs‐erastin and anti‐PD‐L1 treatment was not significantly toxic to the mice (Figure 8F). Subsequent FCM revealed that the combination of Man@pSiNP‐erastin and anti‐PD‐L1 resulted in the strongest decrease in TAM infiltration, suppressed the M2‐like shift, and enhanced the activity of IFN‐*γ* CD8^+^ cells (Figure 8G–I). GZMB is a known marker of effector T cells with cytotoxic activity. IF staining showed that combined treatment with Man@pSiNPs‐erastin and anti‐PD‐L1 increased the cytotoxic activity and proliferation of CD8 T cells (Figure 8J,K), indicating that the combination of Man@pSiNPs‐erastin and anti‐PD‐L1 effectively enhanced antitumor immunity of the body. Figure 8L–O further determines that Man@pSiNP‐erastin and anti‐PD‐L1 combination therapy more strongly increased the expression of immune‐related cytokines, including IFN‐*γ*, TNF‐*α*, IL‐2 and IL‐12, than Man@pSiNPs‐erastin or anti‐PD‐L1 treatment alone. Collectively, combined treatment with Man@pSiNPs‐erastin and anti‐PD‐L1 substantially impeded tumor progression by suppressing TAM infiltration, reducing the M2‐like shift, and enhancing antitumor immunity of the body.

### 2.9 Dissecting the Distinctive Clinical Functional Profiles of xCT Especially TAM‐Derived xCT in HCC

**Figure 9:**
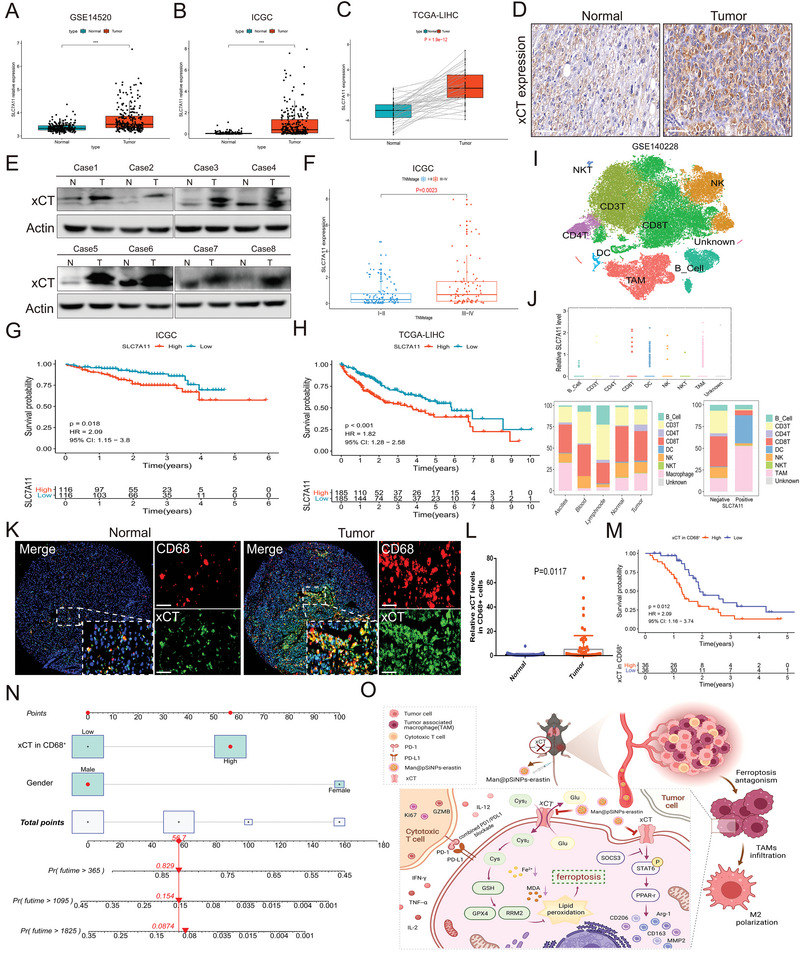
Distinctive clinical functional profiles of xCT and macrophage‐derived xCT in HCC. A–C) Expression of SLC7A11 in tumor tissues and normal tissues in GSE14520 (A), ICGC (B), and TCGA‐LIHC (C). D) IHC staining showing the differences in the expression of xCT in tumor tissue and normal tissue. E) Western blot exhibiting the expression characteristics of xCT in tumor tissues and normal tissues. F) The correlation between SLC7A11 levels and TNM staging in ICGC database. G,H) Survival analysis showing the predictive value of SLC7A11 in survival probability in ICGC (G) and TCGA‐LIHC cohort (H). I) The immune cellular landscape of HCC. J) Expression of SLC7A11 in immune cells and the distribution proportion of immune cells in different tissues. K,L) IF staining showing the proportion of infiltrating CD68+ cells and xCT expression in CD68+ cells in tumor tissues compared to normal tissues. M) Survival analysis indicating a significant survival advantage of HCC patients with low xCT expression in CD68+ cells. N) A nomogram was constructed based on the independent prognostic factors of HCC. O) The overall process and mechanism of this study. Created with Biorender.com. Data in the figure are represented as the means ± SEM. Differences between the groups were evaluated using Student's *t*‐test. *** *p* < 0.001.

The above discoveries highlighted that macrophage‐derived xCT contributes to HCC tumor growth and represses the anti‐PD‐L1 response by restraining TAM ferroptosis and enhancing protumoral polarization. In addition, we further broadened exploration in the clinical functional profiles of xCT, especially TAM‐derived xCT, in HCC. Online databases, including GSE14520, ICGC and TCGA‐LIHC, revealed that the expression of SLC7A11 in tumor tissues was significantly higher than that in normal tissues (**Figure** **9**A–C), and consistent results were obtained through IHC staining (Figure 9D) and western blotting (Figure 9E). A positive correlation between SLC7A11 expression and malignant tumor progression is shown in Figure 9F, and survival analysis indicated that compared to the high expression of SLC7A11, low expression of SLC7A11 led to a higher survival probability in both ICGC and TCGA‐LIHC cohort (Figure 9G,H). Then, we performed scRNA‐sequencing to explore the immune cellular landscape of HCC (Figure 9I), and the expression characteristics of SLC7A11 in immune cells, as well as the distribution of immune cells in different tissues, was also examined. We found that SLC7A11 was highly expressed in TAMs and played a substantial positive impact on TAM infiltration (Figure 9J). The IF staining results further confirmed the increased infiltration of CD68+ macrophages, as well as the stronger expression of xCT in CD68+ macrophages (Figure 9K,L). Survival analysis showed a significant survival advantage in HCC patients with low xCT expression in CD68+ macrophages, indicating that xCT expression in CD68+ macrophages was a potential prognostic factor of HCC (Figure 9M). Comparing the prognostic value of xCT expression in CD68+ macrophages with traditional clinical features, we found that gender and xCT expression in CD68+ macrophages were independent prognostic factors of HCC (Figure S6A, Supporting Information); based on these two prognostic factors, a nomogram was constructed to quantitatively predict the prognosis of HCC patients (Figure 9N). The calibration curves at 0.5 year, 1 year, 2 years, and 3 years showed the consistence between the predicted results of the nomogram and the actual results (Figure S6B–E, Supporting Information). And the results of the ROC curves indicated that predictive performance of the nomogram was superior to a single prognostic factor (Figure S6F–I, Supporting Information). Taken together, these results suggest that xCT exhibited distinct clinical and functional profiles in HCC and that xCT expression in tumors, especially TAM‐derived xCT, could serve as a reliable predictive factor for the prognosis of HCC. And Figure 9O exhibits the overall process and mechanism of this study.

## 3 Discussion

Characterized by a complex and unique molecular pathogenesis and complicated TME, the deterioration of HCC progresses rapidly and significantly restraints the choice of traditional treatment methods, such as hepatic resection and TACE, as well as the efficacy of emerging treatment strategies, such as targeted therapy and immunotherapy.^[^ ^5^, ^39^ ^]^ Accounting for the main component of immune cell infiltration in the TME, TAMs can constitute almost 50% of the tumor mass^[^ ^40^ ^]^ and is featured by functional and phenotypic plasticity, which plays crucial roles in the occurrence and progression of HCC.^[^ ^41^, ^42^ ^]^ Exploring the molecular mechanisms and regulatory pathways affecting the function of TAMs can hopefully achieve targeted precision tumor prevention and treatment. In our study, we revealed the significantly upregulated expression of xCT in TAMs, and xCT knockout in macrophages substantially diminished the infiltration of TAMs, suppressed the M2‐like phenotype shift in HCC tumor tissues, as well as triggering and enhancing the activity of ferroptosis in macrophages and attenuated tumor development and metastasis.

By using different genetic mouse models with systemic or macrophage‐specific xCT knockout, we observed that xCT knockout in macrophages was sufficient to hamper tumor growth and metastasis, demonstrating the striking effect of TAMs on tumor development. Furthermore, we systematically dissected the effect of macrophage‐derived xCT on TAMs alone and the crosstalk between tumor cells and macrophages. We found that xCT knockout impaired macrophage recruitment and polarization by impeding the release of M2‐related inducers and cytokines, including CCL22, IL‐10, MMP9 and MMP2, as well as interfering with the activity of the IL‐4 mediated SOCS3‐STAT6‐PPAR‐*γ* signaling axis. Signal transducer and activator of transcription 6 (STAT6) is an important member of the STAT family, which is of great significance in regulating cell differentiation and cytokine production.^[^ ^43^ ^]^ Phosphorylation of STAT6 is crucial for M2 polarization and can regulate the transcription of M2‐related genes such as Mrc1, Arg1, and Ym1.^[^ ^44^ ^]^ In addition, STAT6 also plays a key role in tumor development and was shown to promote lung cancer progression by triggering an IL‐4 positive feedback loop and increasing M2 myeloid cells.^[^ ^45^ ^]^ STAT6 is also a promoter of PPAR‐*γ* transcription. PPAR‐*γ* is a transcriptional activator involved in regulating cell growth, metabolism and inflammation,^[^ ^46^ ^]^ and is involved in the activation of M2 macrophages.^[^ ^47^ ^]^ SOCS3 is an indispensable tumor suppressor protein in cells whose negative feedback inhibition plays a critical role in weakening cytokine and growth factor signaling. It was confirmed that the loss of SOCS3 made liver cells sensitive to tumor transformation.^[^ ^48^ ^]^ SOCS3 is also an important regulatory factor associated with macrophage function, participating in the regulation of M1/M2 polarization.^[^ ^49^ ^]^ Consistent with these findings, our study unveiled that xCT knockout in macrophages diminished the expression of phospho‐STAT6 and PPAR‐r and enhanced the expression of SOCS3, contributing to inhibition of M2 macrophage polarization.

xCT is also known for its inhibitory effect on ferroptosis, and in recent years, ferroptosis has been gradually considered an adaptive feature that eliminates malignant cells, which is crucial for suppressing tumor development.^[^ ^50^ ^]^ However, previous studies have focused only on the role of ferroptosis in tumor cells. In our study, we found that macrophage‐derived xCT significantly reduced intracellular ferroptosis activity and promoted TAM infiltration, thereby contributing to HCC tumor growth. Further investigation revealed that xCT knockout blocked the upregulation of GPX4 expression in Hepa1‐6 CM‐cultured BMDMs and enhanced ferroptosis‐mediated cell killing in the presence of RSL3. Notably, the expression trend of RRM2 was consistent with that of GPX4 in the presence of xCT knockout, and the RRM2 inhibitor Osalimid also showed a cytotoxic effect similar to that of RSL3. GPX4 is well known as the primary enzyme that prevents ferroptosis, and GPX4 knockout leads to cell death in a pathologically relevant form of ferroptosis.^[^ ^50^ ^]^ RRM2 is considered to be an important component of tumor progression and has been shown to be overexpressed and exhibit oncogenic activity in HCC.^[^ ^51^ ^]^ Importantly, our previous study revealed that RRM2 facilitates tumor immune infiltration of lung adenocarcinoma (LUAD) by inhibiting ferroptotic death.^[^ ^52^ ^]^ Based on previous findings and our research, we reasonably presume that the contribution of xCT knockout to the enhanced induction and activity of ferroptosis in TAMs was associated with GPX4/RRM2 signaling regulation, suggesting RRM2 a promising target gene and Osalimid a potential target drug to activate ferroptosis with macrophages against HCC.

xCT‐mediated TAM ferroptosis and phenotypic polarization significantly changed the TME of HCC and contributed to HCC tumor growth, which highlights the potential of targeting macrophage‐derived xCT to treat HCC and the significance of our study. In addition, clinically, the combination of xCT plus immunotherapy may contribute to a more powerful therapeutic efficacy. Evading immune surveillance is a challenging step in tumor evolution. Previous studies have shown that PD‐L1 can be expressed by TAMs, and its expression is related to the immunosuppressive activity of TAMs, which induces tumor immune escape.^[^ ^53^ ^]^ We unveiled that xCT‐mediated macrophage ferroptosis significantly promoted the expression of PD‐L1, and xCT specific knockout in macrophages combined with anti‐PD‐L1 treatment acquire more substantial antitumor efficacy than xCT knockout in macrophages or anti‐PD‐L1 treatment alone. pSiNPs have been widely used as drug carriers owing to their excellent drug loading capacity, high biocompatibility, and biodegradability.^[^ ^30^, ^54^ ^]^ In our study, we prepared pSiNPs functionalized with mannose and determined that Man@pSiNPs acquired the ability of specifically targeting TAMs with no significant cytotoxicity to the body. Erastin‐loaded Man@pSiNPs exerted a strong antitumoral effect against HCC by triggering and enhancing TAM ferroptosis activity. Our findings suggest that Man@pSiNPs‐erastin is a promising candidate for targeting TAMs against HCC and provides an option for combination therapy with anti‐PD‐L1 to enhance the efficacy of immunotherapy.

When further dissecting the clinical and functional profiles of xCT expression in HCC, it was shown that xCT expression in tumors was higher than that in normal tissues, indicating that xCT is a potential and crucial carcinogenic factor for HCC. The expression of xCT in tumors and CD68+ macrophages was closely related to the prognosis of HCC patients, which provided an effective and reliable predictive tool for the prognosis of HCC patients.

Undeniably, there were also some limitations in the manuscript. The effect of the role of a SOCS3‐STAT6‐PPAR‐*γ* pathway in xCT‐mediated macrophage M2‐like polarization needs to be further confirmed, and we should also further investigate and clarify the specific mechanism in which xCT regulates the SOCS3‐STAT6‐PPAR‐*γ* signaling pathway in the following studies. Besides, we unveiled the ferroptosis induction in the context of macrophage xCT knockout, but the specific factors in the TME influence the ferroptosis within xCT KO TAMs also need to be further explored in the future study.

In conclusion, we provide evidence in the current study that, macrophage‐derived xCT is involved in regulating TAM ferroptosis and polarization in HCC models and confirm that targeting macrophage‐derived xCT is sufficient to promote tumor development and metastasis, which is achieved by inducing ferroptosis and enhancing its activity in macrophages, as well as diminishing TAM recruitment and infiltration, particularly the M2 polarization. Man@pSiNPs‐erastin we developed not only specifically target TAMs to induce intracellular ferroptosis and exert cytotoxic effects but also to strengthen the anti‐PD‐L1 response, which in combination with anti‐PD‐L1 exerts more powerful antitumor efficacy than individual treatment. These results not only highlight the critical role of xCT in TAM function and phenotype but also show a crucial effect of macrophage ferroptosis on HCC progression. The expression of xCT in tumors and in CD68+ macrophages could also serve as an effective predictive factor for the prognosis of HCC. Our study reveals that targeting xCT‐mediated macrophage ferroptosis and protumor phenotype polarization is effective against HCC and highlights its clinical significance in targeted antitumor therapy and immunotherapy.

## 4 Experimental Section

### Cell Culture

Hepa1‐6 (murine hepatoma cell line), LLC (murine Lewis lung carcinoma), CT26 (mouse colon cells), 4T1 (mouse breast cancer cells), and RAW264,7 cells were purchased from the American Type Culture Collection (ATCC) (Manassas, VA, USA). Hepa1‐6, LLC, CT26, and 4T1 cells were cultured in DMEM (Gibco, NYC, USA) containing 10% fetal bovine serum (FBS) (Gibco, NYC, USA) and 1% penicillin/streptomycin (Gibco, NYC, USA). RAW264.7 cells were cultured in RPMI 1640 medium (Gibco, NYC, USA) containing 10% FBS and 1% penicillin/streptomycin. The cells were grown in a humidified cell incubator at 37 °C in a 5% CO_2_ atmosphere.

### Mice

xCT^−/−^ mice (denoted as xCT KO), xCT^flox/flox^ mice (denoted as wild‐type (WT) or xCT^f/f^) and LysMCre mice were provided by Cyagen Biosciences Inc. xCT^f/f^ mice were crossed with LysMCre mice to generate macrophage‐specific xCT‐KO mice (denoted as xCT^lyz2cre^). Mice were genotyped from tail biopsy using the primers listed in the Supporting Information. All mice were on a C57BL/6 background and were maintained under pathogen‐free conditions. Mice aged 8 weeks were used for animal experiments in this study. All experiments involving animals were conducted according to the ethical policies and procedures approved by Experimental Animal Welfare Ethics Review Committee of Zhejiang University, China.

### Extraction of Bone Marrow‐Derived Macrophages (BMDMs)

The mice were euthanized by cervical dislocation under isoflurane anesthesia. Then, the mice were soaked and disinfected in a sufficient amount of 75% ethanol for 5 min, and the entire leg bone was exposed along the direction of muscle growth. The femurs and tibias were flushed with 5 mL of serum‐free 1640 medium to obtain bone marrow cells. After centrifuging the suspension at 1500 rpm for 5 min, the supernatant was discarded, 1 mL of red blood cell lysis buffer was used to resuspend the bone marrow‐derived cells, and after standing for 5 min the suspension was centrifuged again at 1500 rpm for 5 min and the supernatant was discarded. Then, the obtained cells were cultured in RPMI 1640 medium containing 20 ng mL^−1^ M‐CSF and 10% FBS for 7 days to harvest M‐CSF‐differentiated macrophages for subsequent experiments.

### Conditioned Medium (CM) Preparation

Hepa1‐6 cells were cultured in a 37 °C constant temperature incubator containing 5% CO_2_ and grown to 80% confluence. Then, the cells were washed with PBS twice and incubated in serum‐free medium for 24 h. Subsequently, CM was collected and filtered through a 0.22 µm membrane sterile filter and stored at −20 °C for subsequent experiments.

### Cell Viability Assay

The Cell Counting Kit‐8 (CCK‐8) assay was used to measure cell viability. BMDMs were cultured in a 96‐well plate (5 × 10^4^ cells/well) with 100 µL of 1640 medium in each well treated with different concentrations of erastin or RSL3 and grown in a 37 °C constant temperature incubator containing 5% CO_2_. Cell Counting Kit‐8 (CCK‐8) reagent (20 µL, MedChemExpress, New Jersey, USA) was added to each well according to the manufacturer's instructions and incubated for 2 h prior to measuring the OD450 nm with a microplate reader (Bio‐Rad, Berkeley, USA). Each group was replicated with three wells and each experiment was repeated at least thrice.

Then the BMDMs were cultured in Hepa1‐6 CM or normal medium for 48 h and then plated in a 6‐well plate (1 × 10^6^ cells/well) and treated with 2 µm erastin or 40 nm RSL3 with or without 4 µm ferrostatin‐1 (Fer‐1) for 0–48 h, followed by the count of living cells using a Countess 3 FL automated cell counter (Lot#AMQAX2000, ThermoFisher, USA). And the LIVE/DEAD Viability/Cytotoxicity Kit (Lot#1 976 809, Invitrogen, CA, USA) was also used to detect the number of living dead cells.

### Transwell Migration Assay

Transwell assays were performed to evaluate the migration of BMDMs from xCT^f/f^ mice and xCT^lyz2cre^ mice. To exclude the influence of proliferation on migration, BMDMs were treated with mitomycin C (10 µg mL^−1^) for 1 h to repress cell proliferation prior to the transwell assay. Then, the cells were seeded into the upper chambers of the 6‐well Transwell plate in 1 mL of serum‐free RPMI 1640 with a density of 2 × 10^5^ cells/100 µL, and complete RPMI 1640 containing 10% FBS was added to the lower chambers. After being incubated at 37 °C for 48 h, nonmigrated cells from the upper side of the chamber were removed with cotton swabs, and migrated cells were fixed with 4% paraformaldehyde for 30 min and then stained with 0.1% crystal violet for another 30 min. Five visual fields of migrated cells were visualized and counted under an optical microscope.

### Immunofluorescence (IF) Staining

Cells were cultured on round coverslips in 6‐well plates, fixed with 4% paraformaldehyde for 30 min and then incubated with 0.3% Triton X‐100 for 15 min to enhance cell membrane permeability. Subsequently, the cells were incubated in 5% bovine serum albumin for 40–60 min. After being washed with PBS 3 times, the cells were incubated with the corresponding primary antibodies overnight at 4 °C, followed by an additional 1‐h incubation with the fluorescently labeled secondary antibody and 15 min of incubation with DAPI at room temperature in the dark. A fluorescence microscope (Nikon, Japan) was used to visualize the fluorescence staining, and ImageJ software (version 1.8.0) was used to quantitatively analyze the staining results.

### Murine Xenograft Models

The mice were injected subcutaneously with 200 µL Hepa1‐6 cell suspension (5 × 10^6^ cells/ mL) in the lower ventral side and then placed in the same sterile environment. When a subcutaneous tumor was visible to the naked eye (nearly 2 mm) (approximately one week after implantation), the tumor size was measured every 3 days. The tumor volume (TV) was calculated according to the following formula: TV (mm^3^) = *L* × *W* ^2^ × 0.5. At least 5 mice were used per group, and each experiment was repeated at least thrice.

The construction of hydrodynamic tail vein injection models of HCC was performed as described.^[^ ^23^, ^24^, ^25^ ^]^ In brief, the density of Hepa1‐6 cell suspension was adjusted to 5 × 10^5^ cells/ mL, and 2 mL of Hepa1‐6 cell suspension was injected into the tail vein of mice in 5–7 s. After 3 weeks of injection, the mice were euthanized by cervical dislocation under isoflurane anesthesia and the tumors were obtained for subsequent experiments.

### Fluorescence‐Activated Cell Sorting (FACS) and TAM Isolation

Hepa1‐6 tumor‐bearing mice were euthanized by cervical dislocation under isoflurane anesthesia, and the tumors were obtained. The tumors were chopped and incubated with 1 mg mL^−1^ collagenase IV (Sigma, USA) and 100 µg mL^−1^ DNase I (Roche, Basel, Switzerland) for 1 h, followed by filtration through a cell strainer (70 µm) to obtain single‐cell suspensions. After lysing the red blood cells using RBC lysis buffer, the obtained cells were washed twice with staining buffer (PBS supplemented with 2% FBS and 1 mm EDTA), resuspended in FACS buffer and incubated with the relevant antibody cocktails on ice for 20 min in the dark. Intracellular staining was performed with permeabilization buffer (eBioscience, USA) and PE‐conjugated primary antibodies. Cell viability was evaluated with 7‐amino actinomycin D (BD Biosciences, USA). FACSCalibur Flow Cytometry Systems were used for sample analysis, and FlowJo software (version 10.6.2) was used for FACS data analysis. A FACSARIA III flow cytometer (BD Biosciences, USA) was used for TAM sorting. The gating strategy was performed as described^[^ ^26^, ^27^ ^]^ that from all viable CD45+ cells, TAM was identified as F4/80+CD11b+. TAMs were sorted into RPMI 1640 containing 10% FBS for subsequent experiments.

### HCC Lung Metastasis Mouse Model Construction

Hepa1‐6 cells were resuspended in PBS (≈1 × 10^6^ cells/mouse) and injected as slowly as possible into the caudal veins of xCT^f/f^ mice and xCT^lyz2cre^ mice to construct HCC lung metastasis mouse models. Bioluminescence imaging in vivo was performed to examine the progression of lung metastasis on the 7th, 14th, and 21st days after injection. Then, the mice were euthanized by cervical dislocation under isoflurane anesthesia, and the lungs were removed for observation and histological analysis.

### Histological Analysis

The lungs were washed, fixed in 4% paraformaldehyde for 24 h and embedded in paraffin after being dehydrated. The lung tissues were sectioned into slices (4 × 4 µm), and deparaffinization and rehydration were performed. Hematoxylin and eosin (H&E) staining was employed for histological analysis.

### Transmission Electron Microscopy (TEM)

BMDMs from xCT^f/f^ mice and xCT^lyz2cre^ mice were cultured with Hepa1‐6 CM, treated with 20 µm RSL3 and cultured for 24 h. Subsequently, BMDMs were washed 3 times with 1 × PBS and fixed with 2.5% ice‐cold glutaraldehyde solution (0.1 m phosphate buffer, pH 7.4) at 4 °C for 12 h. Then, the cell samples were treated to meet the requirements of TEM as previously described.^[^ ^28^ ^]^ A JEM‐1400Flash transmission electron microscope (JEOL Ltd., Japan) was used to capture ultrastructural images of the cells at 80 kV.

### Western Blot (WB) Analysis

RIPA lysis buffer containing 1% protease and phosphatase inhibitors was used to extract proteins from Hepa1‐6 tumor cells co‐cultured BMDMs. Protein quantification was carried out using a BCA protein assay kit. Then, 10–12% sodium dodecyl sulfate‐polyacrylamide gel electrophoresis (SDS‐PAGE) was performed to resolve the protein samples, and a polyvinylidene fluoride (PVDF) membrane was used for protein electrotransfer, followed by membrane blocking in 5% milk for 45 min. After that, the membrane was incubated with the corresponding primary antibodies at 4% overnight and incubated with HRP‐conjugated secondary antibodies at room temperature for another 2 h. The protein bands in the membrane were examined and visualized on an iBright FL1500 Imaging System. Details of the antibodies used in the study can be seen in the Supporting Information.

### Quantitative Reverse Transcription‐Polymerase Chain Reaction (qRT‐PCR)

Total RNA was extracted from treated BMDMs and TAMs with TRIzol reagent by a Direct‐zol RNA MiniPrep Kit (Zymo Research), following with the reverse transcription of 2 µg of total RNA to cDNA by the RevertAid First Strand cDNA Synthesis kit (Thermo Scientific). Next, qRT‐PCR was performed using a SYBR Green PCR Master Mix (Thermo Scientific). Relative mRNA expression (fold change) was calculated using the 2‐ΔΔCT methodology and normalized to 18S mRNA levels. The sequences of the primers used in this study are provided in the Supporting Information. Each experiment was conducted independently at least three times.

### Porous Silicon Nanoparticle (pSiNP) Preparation

pSiNPs were prepared by the electrochemical etching method as previously described.^[^ ^29^, ^30^ ^]^ Briefly, boron‐doped p^++^‐type Si was electrochemically etched in an aqueous solution of 48% hydrofluoric acid (HF) and absolute ethanol (3:1, v/v) in a Teflon cell. A constant current density of 200 mA cm^−2^ was carried out for 150 s. The porous layer on the substrate was then removed in a 3.3% aqueous HF solution in ethanol by a constant current of 4.5 mA cm^−2^ for 90 s, followed by fracture in ultrapure water by ultrasonication overnight. Then, the solution was centrifuged at 2700 × g for 2 min to remove the largest particles and subsequently centrifuged at 22 000 × g for 30 min to remove the smallest particles. pSiNPs were then collected and dispersed in absolute ethanol.

### pSiNP Functionalization with Mannose

First, 0.03 mmol of mannose (P‐{n‐(2‐ethoxy‐3,4‐dioxocyclobut‐1‐enyl) amino]}phenyl‐*α*‐D‐mannopyranoside), 0.04 mmol of aminopropyltriethoxysilane (APTES), 11 µL (0.07 mmol) of triethylamine and 2 mL of dried THF were added to a round bottom flask, mixed and stirred at room temperature under nitrogen overnight. The freshly prepared pSiNPs were centrifuged in absolute ethanol at 22 000 × g for 30 min. 30 mg pSiNPs and APTES‐Man were dispersed in 3 mL of DMF and reacted at 60 °C under nitrogen overnight. The mixture was then centrifuged at 22 000 × g for 30 min and washed 5 times with absolute ethanol to collect Man@pSiNPs. Man@pSiNPs (20 mg) was dispersed in 10 mL of absolute ethanol.

### Identification of Differentially Expressed Genes (DEGs) between TAMs in Hepa1‐6 Tumors and BMDMs

Total RNA sequencing of TAMs in Hepa1‐6 tumors and BMDMs was performed using a TruSeq Stranded Total RNA Library Preparation Kit with Ribo‐Zero Gold (Illumina) as described previously.^[^ ^31^ ^]^ The limma R package was used to identify DEGs between TAMs in Hepa1‐6 tumors and BMDMs with the following cutoff values: absolute log twofold change (FC) > 1 and adjusted *p* value <0.05.

### Functional Enrichment Analysis

KEGG analysis was performed on the Dr.Tom website (https://biosys.bgi.com/) to examine the enriched regulatory signaling pathways of the DEGs. Results with an adjusted *p* < 0.05 were considered statistically significant.

### Statistical Analysis

Statistical analysis was carried out using GraphPad Prism (version 8.0) software and the R package. The statistical significance for comparisons of two groups was evaluated using the Student's *t*‐test. Pearson correlation analysis was performed to calculate the corresponding *R* and *p* values of the scatter plot. One‐way ANOVA followed by Tukey's multiple comparisons test were conducted to compare the differences among multiple groups. All data are presented as the mean ± standard error of mean (SEM). *p* < 0.05 was considered statistically significant. Statistical significance is denoted (no significance [ns], *p < 0.05; **p < 0.01; ***p < 0.001; ****p < 0.0001) in the figures and figure legends. All experiments were independently repeated at least thrice.

## Conflict of Interest

The authors declare no conflict of interest.

## Author Contributions

B.T. and J.Z. contributed equally to this work. J.J., M.C. and Z.Z. were involved in the conception, design and supervision of the study. B.T. and J.Z. were involved in the development of methodologies, acquisition, analysis, and interpretation of data. Y.W., W.C., S.F. and W.M. were involved in the performance of experiments, data collection and analysis. Z.X., Y.Y. and Q.W. were involved in reagents, materials and analysis tools contribution. B.T. and J.Z. finished the manuscript writing. J.J. and M.C. edited the paper.

## Supporting information

Supporting Information

## Data Availability

The data used to support the findings of this study are available from the corresponding author upon request.
