# Supplementary material for: Targeted xCT‐mediated Ferroptosis and Protumoral Polarization of Macrophages Is Effective against HCC and Enhances the Efficacy of the Anti‐PD‐1/L1 Response
Source: Adv Sci (Weinh). 2022 Nov 28;10(2):2203973. doi: 10.1002/advs.202203973 (PMC9839855; doi:10.1002/advs.202203973)
Supplement: Supplementary file 1 — Supporting Information [file ADVS-10-2203973-s001.pdf]

Supplementary figure legends

Figure S1. SLC7A11 knockout in the systemic xCT-KO mice and the $xCT^{LysMcre}$ mice. (A) Genotyping analysis showing the confirmation of the systemic xCT-KO mice. **(B)** Genotyping analysis showing the confirmation of the $xCT^{LysMcre}$ mice. **(C)** Western blot analysis showing the expression of xCT in different tissues of mice of different genotypes.

Figure S2. Flow cytometry analysis of blood monocytes in $xCT^{f/f}$ and $xCT^{lyz2cre}$ mice. (A) Representative pictures of gating strategy to identified CD11b+ Ly6C as monocytes. **(B)** The frequency of Ly6Chigh, Ly6Chi and Ly6Clow between $xCT^{f/f}$ and $xCT^{lyz2cre}$ mice.

Figure S3. Correlation of immune cell infiltration in the TME and xCT expression from ICGC database.

Figure S4. Construction of hydrodynamic tail vein injection models of HCC. (A) In vivo bioluminescence imaging showing the tumor growth in $xCT^{f/f}$ and $xCT^{lyz2cre}$ mice. **(B)** HE staining exhibiting the pathological characteristics of heart, lung, kidney and spleen.

Figure S5. Changes in tumor and organs of mice in response to different treatments. (A) The changes in tumor volume in response to different treatments. **(B)** Changes in body weight in response to different treatments. **(C)** HE staining showing pathological changes of the organs including heart, liver, spleen, lung and kidney in response to different treatments.

Figure S6. Predictive performance of the nomogram. (A) The independent prognostic predictive value of xCT expression in CD68+ cells compared to traditional clinical features. **(B-E)** Calibration curves of the nomogram at 0.5 year, 1 year, 2 years, and 3 years. **(F-I)** ROC curves showing the predictive performance of the nomogram compared to a single independent predictor.

Supplementary figures

Figure S1

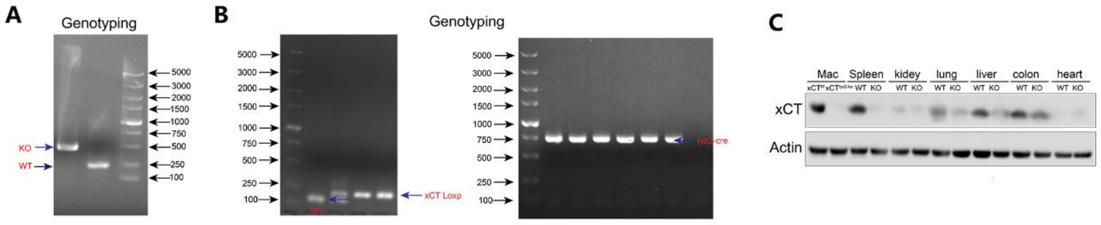

Figure S2

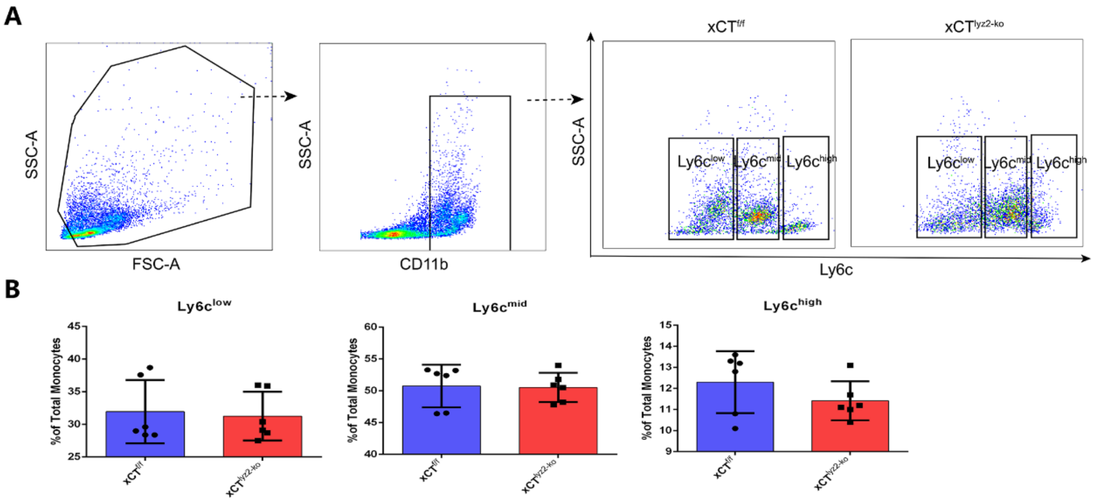

Figure S3

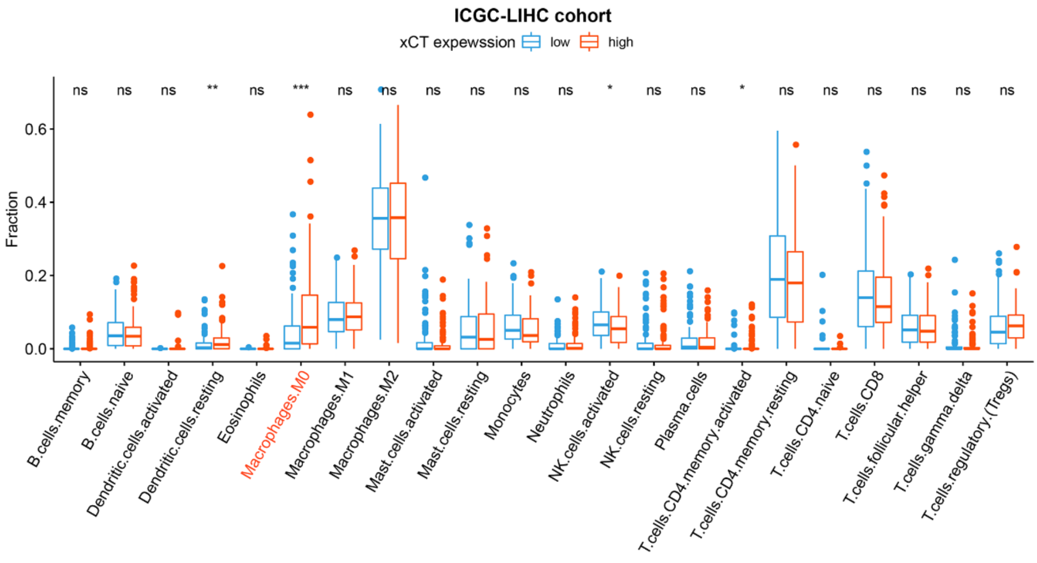

Figure S4

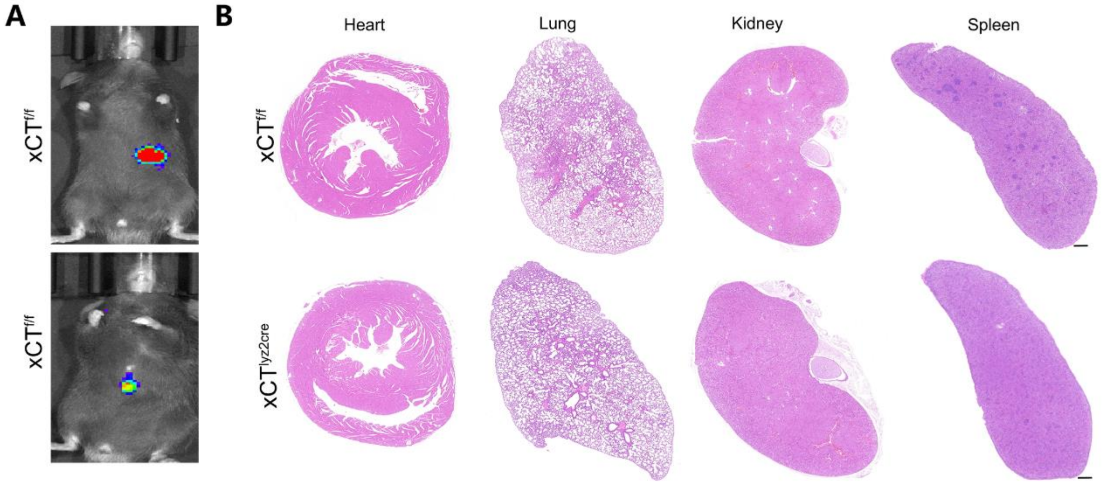

Figure S5

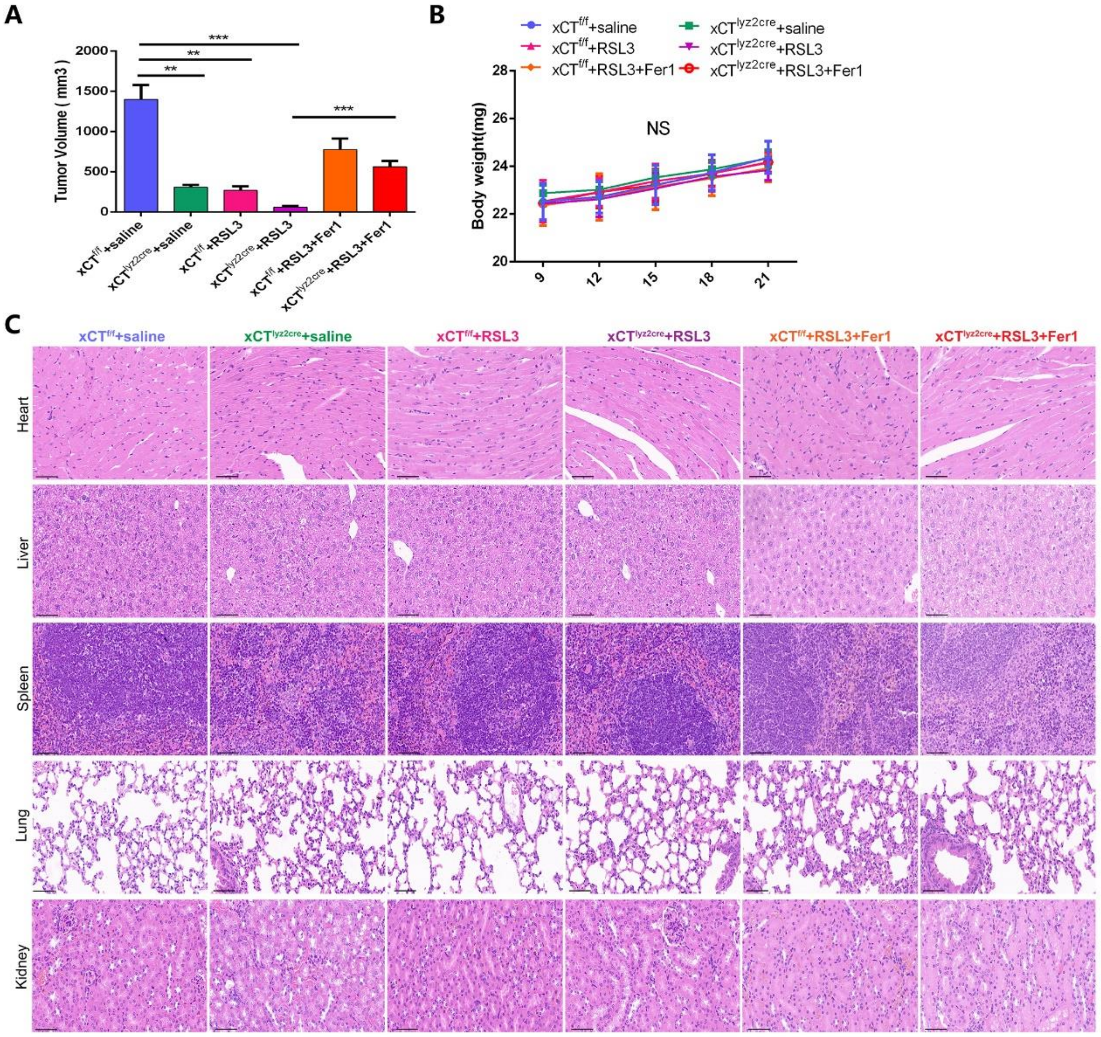

Figure S6

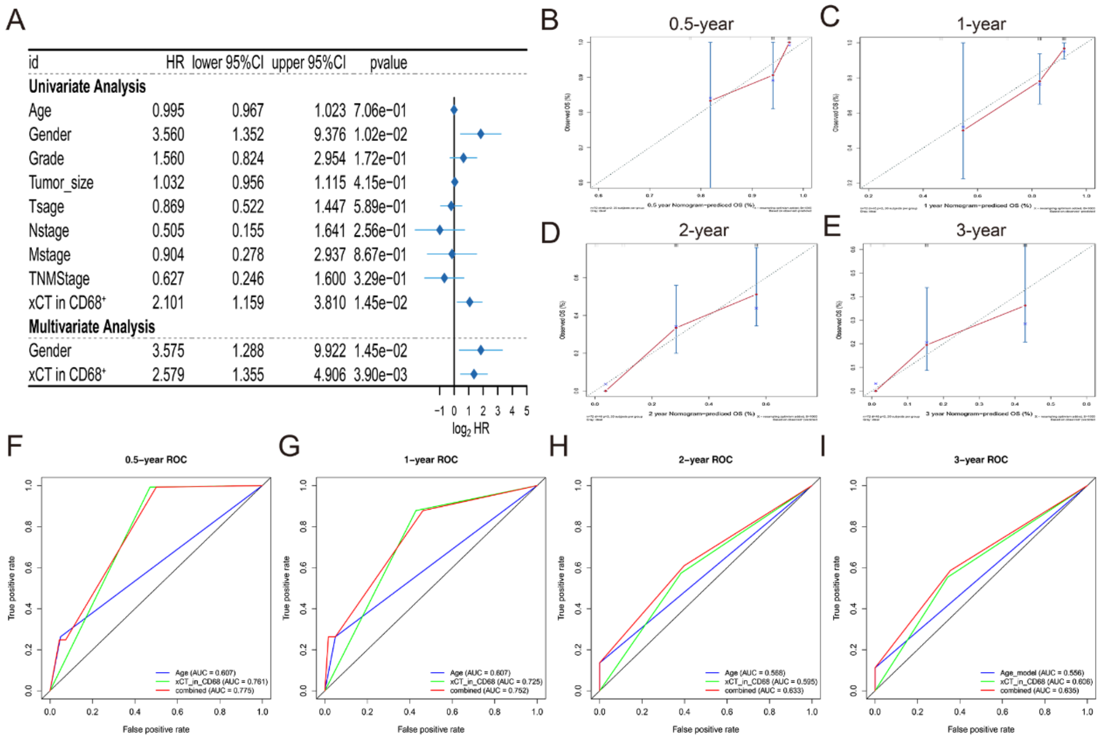
A
id
HR
lower 95%CI
upper 95%CI
pvalue
Univariate Analysis
Age 0.995 0.967 1.023 7.06e−01
Gender 3.560 1.352 9.376 1.02e−02
Grade 1.560 0.824 2.954 1.72e−01
Tumor_size 1.032 0.956 1.115 4.15e−01
Tsage 0.869 0.522 1.447 5.89e−01
Nstage 0.505 0.155 1.641 2.56e−01
Mstage 0.904 0.278 2.937 8.67e−01
TNMStage 0.627 0.246 1.600 3.29e−01
xCT in CD68+ 2.101 1.159 3.810 1.45e−02
Multivariate Analysis
Gender 3.575 1.288 9.922 1.45e−02
xCT in CD68+ 2.579 1.355 4.906 3.90e−03
log2 HR
B
0.5-year
C
1-year
D
2-year
E
3-year
F
0.5−year ROC
Age (AUC = 0.607)
xCT_in_CD68 (AUC = 0.761)
combined (AUC = 0.775)
G
1−year ROC
Age (AUC = 0.607)
xCT_in_CD68 (AUC = 0.725)
combined (AUC = 0.752)
H
2−year ROC
Age (AUC = 0.568)
xCT_in_CD68 (AUC = 0.595)
combined (AUC = 0.633)
I
3−year ROC
Age_model (AUC = 0.556)
xCT_in_CD68 (AUC = 0.606)
combined (AUC = 0.635)
True positive rate
False positive rate

1.1 Genotyping primers

Name		Sequence
xCT floxed	Forward	TCTCAAAAGCATCATCCAGG
	Reverse-1	GCAAGGCTCCCTAATGATTTCT
	Reverse-2	CCACCACCTCATACTAAGACAAAG
LysMcre	Forward	CCCAGAAATGCCAGATTACG
	Reverse	CTTGGGCTGCCAGAATTTCT
xCT loxp	Forward	AATCTCACACGGCATGTACTCTCTTCTT
	Reverse	GCTGACCTATTATTTGAGTAACACCACCA

1.2 Antibodies

Name	Dilution	Species	Manufacture	Cat, No.	Application
xCT	1:1000	Rabbit	Abcam	ab37185	Western blot
	1:200				IF
PD-L1	1:1000	Rabbit	Cell Signaling Technology	#13684	Western blot
	1:400			#41726	IF
CD31	1:800	Rabbit	Cell Signaling Technology	#3528	IF
PCNA	1:400	Rabbit	Cell Signaling Technology	#13110	IF
Ki67	1:10000	Mouse	Cell Signaling Technology	#9449	IF
CD68	1:400	Rabbit	Cell Signaling Technology	#97778	IF
GZMB	1:400	Rabbit	Cell Signaling Technology	#17215	IF
F4/80	1:400	Rabbit	Cell Signaling Technology	#30325	IF
CD206	1:1000	Rabbit	Cell Signaling Technology	#24595	Western blot
	1:400				IF
GPX4	1:1000	Rabbit	Cell Signaling Technology	#59735	Western blot
MMP2	1:1000	Rabbit	Cell Signaling Technology	#40994	Western blot
Arginase-1	1:1000	Rabbit	Cell Signaling Technology	#93668	Western blot
	1:50				IF
CD163	1:1000	Rabbit	Cell Signaling Technology	#25121	Western blot
STAT6	1:1000	Rabbit	Cell Signaling Technology	#5397	Western blot
P-STAT6	1:1000	Rabbit	Cell Signaling Technology	#56554	Western blot

PPAR-b	1:1000	Rabbit	Abcam	ab178866	Western blot
PPAR-α	1:1000	Rabbit	Abcam	ab126285	Western blot
PPAR-γ	1:1000	Rabbit	Cell Signaling Technology	#2435	Western blot
SOCS3	1:1000	Rabbit	Cell Signaling Technology	#52113	Western blot
P-AKT	1:1000	Rabbit	Cell Signaling Technology	#4060	Western blot
AKT	1:1000	Rabbit	Cell Signaling Technology	#4691	Western blot
RRM2	1:1000	Rabbit	Cell Signaling Technology	#65939	Western blot
β-Actin	1:4000	Mouse	Abcam	ab8226	Western blot
Goat anti Rabbit IgG-HRP	1:10000	Goat	ABclonal Technology	AS014	Western blot
Goat anti Mouse IgG-HRP	1:10000	Goat	ABclonal Technology	AS003	Western blot

1.3 FACS antibodies

Name	Fluorophore	Clone	Manufacture	Cat, No.
F4/80	PE	BM8	Biolegend	123110
CD206	FITC	C068C2	Biolegend	141703
CD45	APC	30-F11	Biolegend	103112
CD11b	FITC	M1/70	Biolegend	101205
CD16/32	FITC	93	Biolegend	101306
CD8	FITC	5H10-1	Biolegend	100803
CD3	APC	17A2	Biolegend	100236
CD4	PE	GK1.5	Biolegend	100408

1.4 qRT-PCR primers

Name		Sequence	Supplier
h-SLC7A11	Forward	GCCCAAGGGGAGACACAAAA	
	Reverse	TGTGCGACTCATAGAATAACTGC	
m-actin	Forward	TGAGCTGCGTTTTACACCCT	
	Reverse	GCCTTCACCGTTCCAGTTTT	

h-actin	Forward	CATGTACGTTGCTATCCAGGC	TSINGKE Biological Technology, Beijing, China.
	Reverse	CTCCTTAATGTCACGCACGAT	
h xCT	Forward	TGCCCTTTCCCTCTATTCGG	
	Reverse	AACCCGCGGTACTCTTTCTTT	
m-xCT	Forward	TACGAAAGTCGCCAGGTCTCT	
	Reverse	TTCCACCCAGACTCGAACAA	
m-MRC1	Forward	GGCTGATTACGAGCAGTGGA	
	Reverse	ACATGCCAGGGTCACCTTTC	
m-ARG1	Forward	ACATTGGCTTGCGAGACGTA	
	Reverse	ATCACCTTGCCAATCCCCAG	
m-MMP2	Forward	CAAGTTCCCCGGCGATGTC	
	Reverse	TTCTGGTCAAGGTCACCTGTC	
